# Modulation of pulmonary IL-21 expression during latent TB and *M*. *tuberculosis*/SIV coinfection

**DOI:** 10.1172/jci.insight.199217

**Published:** 2026-05-05

**Authors:** Vinay Shivanna, Renee D. Escalona, Colin Chuba, Shashi Prakash Singh, Ahmed A. Moustafa, J. Quincy Brown, Chenyao Xiao, Sangkyu Kim, Edward J. Dick, Smriti Mehra, Mirko Paiardini, Riti Sharan

**Affiliations:** 1 Southwest National Primate Research Center, Texas Biomedical Research Institute, San Antonio, Texas, USA.; 2 Department of Medicine, Tulane Center for Aging, Tulane University Health Science Center, New Orleans, Louisiana, USA.; 3 Department of Zoology and Entomology, Faculty of Science, Capitol University, Cairo, Egypt.; 4 Tulane School of Science & Engineering, Tulane Cancer Center, Tulane University School of Medicine, New Orleans, Louisiana, USA.; 5 Division of Microbiology & Immunology, Emory National Primate Research Center, and; 6 Department of Pathology and Laboratory Medicine, Emory University School of Medicine, Atlanta, Georgia, USA.

**Keywords:** AIDS/HIV, Immunology, Infectious disease, Immunotherapy, Th1 response, Tuberculosis

## Abstract

Tuberculosis (TB) and HIV coinfection remains a major global health challenge, with limited understanding of how these pathogens affect local immune responses in the lungs. This study is the first to our knowledge to investigate the modulation of IL-21 during LTBI and *M*. *tuberculosis*/SIV coinfection in nonhuman primates (NHP). We show that IL-21 expression, predominantly derived from CD4^+^ T cells, is significantly reduced in lungs of *M*. *tuberculosis*/SIV coinfected macaques, especially in the absence of cART. Although cART and cART with 3HP partially restore IL-21–producing CD4^+^ T cells, levels remain below those in LTBI, indicating ongoing immune impairment. Spatial transcriptomic analysis suggests localized alterations in immune signaling, including differences in STAT1- and STAT3-associated transcriptional profiles and reduced *M*. *tuberculosis*–specific IFN-γ responses in coinfected animals. Together, our findings indicate that IL-21–producing CD4^+^ T cells are selectively and persistently impaired in the lungs during *M*. *tuberculosis*/SIV coinfection, despite antimicrobial and antiviral therapy. These results highlight a compartment-specific deficit in immune reconstitution and suggest that IL-21–associated pathways may warrant further investigation as potential targets for host-directed therapeutic strategies.

## Abstract 2

<section data-scroll-anchor=”true” data-testid=”conversation-turn-6” data-turn=”assistant” data-turn-id=”request-WEB:3e647ddf-4cba-4366-80a9-251061807043-2” dir=”auto”> Lung immune signal IL-21 drops with TB and HIV co-infection; treatment partly restores immunity, but imbalance persists, suggesting IL-21 supplementation could improve recovery and infection control. </section>

## Graphical abstract

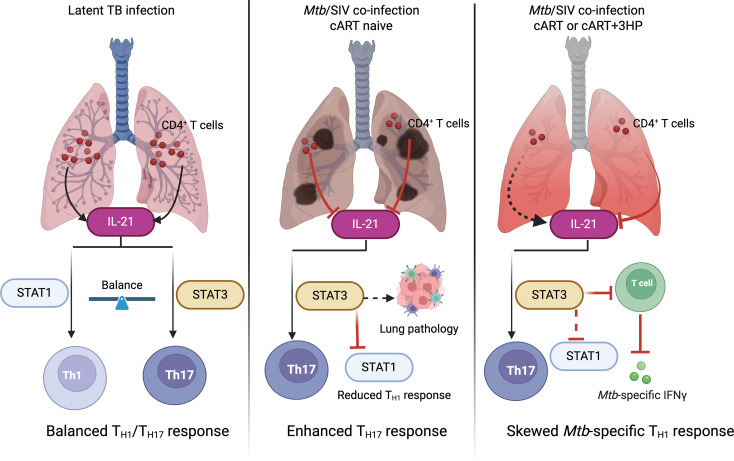

## Introduction

Tuberculosis (TB) is a leading cause of death among people living with HIV (PLHIV). Despite progress in treatment regimens and a reduction of 8.7% in the TB incidence rate from 2015 to 2022, TB remains the leading infectious disease killer in the world (1). Infection with *M*. *tuberculosis* manifests itself as a latent TB infection (LTBI) in most immunocompetent individuals, wherein the bacilli are contained in a dormant state within an immune microenvironment termed granuloma (2–5). Coinfection with HIV weakens the lung immune response, disrupts bacterial containment, triggering release of bacilli and reactivation of LTBI (6–8). At present, there is no clear explanation for why some but not all patients with LTBI experience debilitating reactivation of TB following HIV infection. Though treatment for HIV is available in the form of combinatorial antiretroviral therapy (cART), its administration to TB/HIV coinfected individuals can result in LTBI reactivation within days to weeks of initiation (9, 10). The mechanisms of why effective cART (resulting in full suppression of HIV viremia) is not able to eliminate the risk of reactivation of LTBI are poorly understood (11) but may be due to insufficient reconstitution of protective, *M*. *tuberculosis*–specific Th1, CD4^+^ T effector memory cells (CD4^+^ Tem), and failure to control immune activation in lung (12). Additionally, administration of antitubercular therapy concurrently with cART reduces reactivation better than cART among individuals with LTBI (13, 14). However, long-term sterilization and immune reconstitution in lungs has not been shown in these individuals. Hence, host-directed therapies that may reconstitute these immune deficits long-term after treatment cessation could likely reduce ability of *M*. *tuberculosis* to reactivate from latency.

IL-21, a cytokine mainly produced by CD4^+^ T and NK cells, regulates Th17 expansion through enhanced expression of IL-23R, and Th1 immune responses through *STAT1* activation, which are compromised following HIV infection (15–18). In mice, IL-21 plays a role in regulating T cell differentiation and enhancing T cell cytokine production in *M*. *tuberculosis* infection (19, 20). However, very little data exist on IL-21 modulation in *M*. *tuberculosis* and *M*. *tuberculosis*/SIV in nonhuman primates (NHPs). Recent studies show that administration of IL-21–IgFc fusion protein to SIV-infected, cART-treated rhesus macaques (RMs) reduces inflammation and preserves Th17 in gut mucosa (19–22); however, whether there are similar improvements in lung-localized T cell responses has not been determined. Notably, IL-21 is emerging as a promising candidate for host-directed therapy (HDT) due to its ability to modulate *STAT1* driven Th1 responses and enhance host defense mechanisms. As a cytokine produced primarily by CD4^+^ T cells, IL-21 plays a crucial role in promoting the function of NK cells, CD8^+^ cytotoxic T lymphocytes, and B cells, thereby augmenting both innate and adaptive immunity. In the context of infectious diseases or cancer, IL-21–based HDT can potentially overcome immune exhaustion and improve pathogen or tumor clearance by restoring T cell activity and promoting a more effective immune microenvironment. Moreover, its ability to synergize with other therapies highlights its potential in combination treatments aimed at enhancing overall therapeutic outcomes while minimizing pathogen resistance. However, further clinical studies are needed to determine the optimal dosing and minimize possible inflammatory side effects.

The RM model of low-dose TB infection, followed by subsequent SIV infection, is an ideal model for investigating the IL-21 biology due to its physiological and biological similarities to humans. We used chromogenic multiplex staining and Xenium spatial transcriptomics to assess how cART alone or with anti-TB therapy (cART+3HP) affects IL-21 biology in the lungs of *M*. *tuberculosis*/SIV coinfected versus latent *M*. *tuberculosis*–infected RMs. CD4^+^ T cells positive for IL-21 production were enumerated in lung formalin fixed paraffin embedded (FFPE) tissue sections from LTBI, cART naive, cART-treated and cART+3HP-treated RMs (11, 12, 23). We further performed high-resolution, imaging-based in situ spatial profiling (Xenium) of the lung tissue sections from a single macaque from each study group to measure the transcript expression of 176 RNA targets specific to Th1, Th17, and immune activation response. We observed a trend toward Th17 polarization after SIV coinfection in our model aligning with humans where CD4^+^ Th cells with Th1/17 polarization have a preferential role as a long-term reservoir for HIV-1 infection during antiretroviral therapy (24). The results not only shed light on the intricate dynamics of IL-21, a cytokine that plays a pivotal role in immune responses to TB and HIV, but also highlight how conventional anti-HIV and anti-TB treatment regimens affect its modulation within the pulmonary compartment.

## Results

### Evaluation of clinical outcomes in LTBI and M. tuberculosis/SIV coinfected RMs treated with cART or cART+3HP.

**Figure 1:**
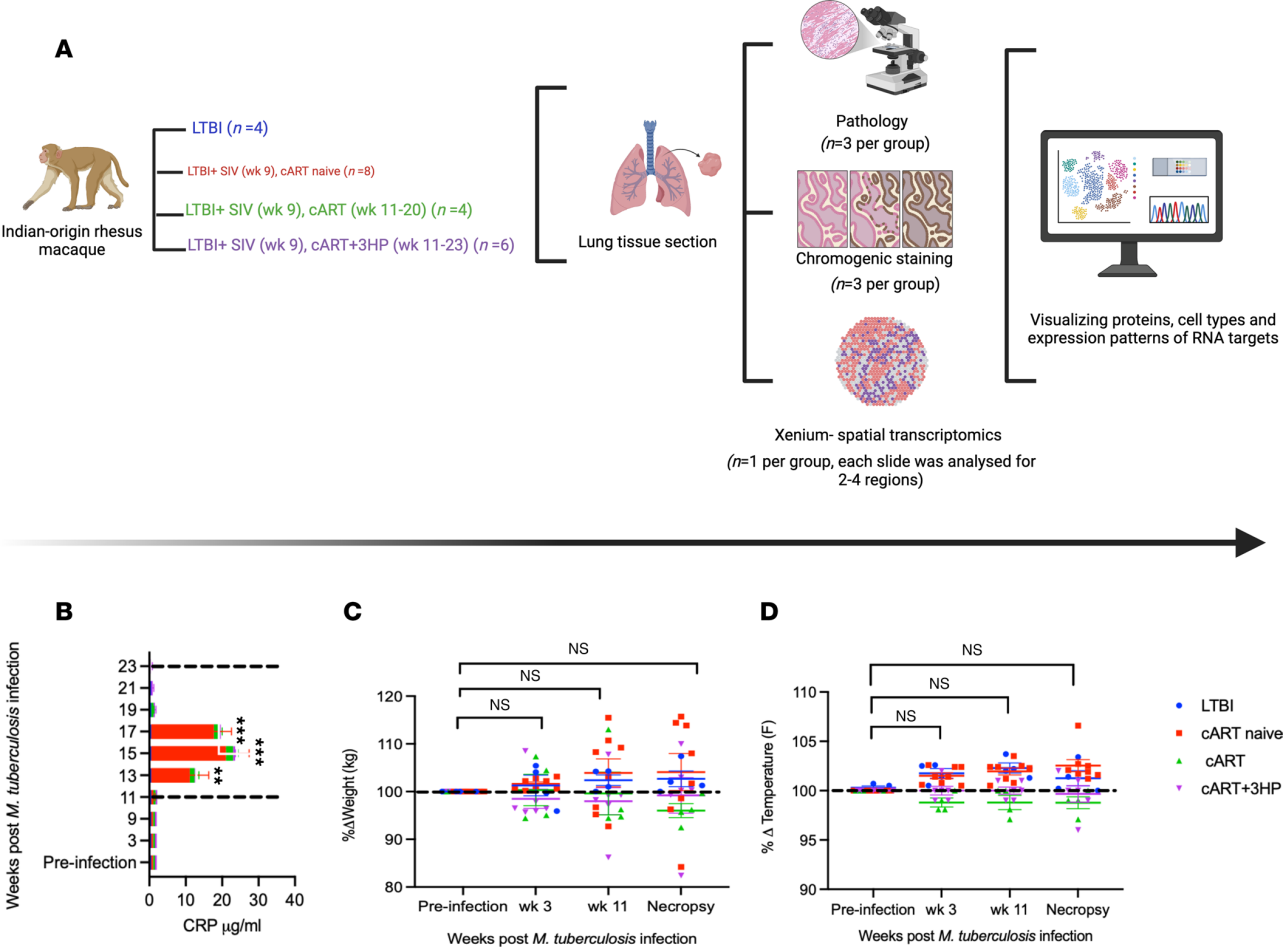
Clinical outcome in LTBI, cART naive, cART-, and cART+3HP–treated RMs. (**A**) Study design outlining the study groups and the downstream pipeline of staining of tissues and analysis. (**B**) Serum CRP levels were measured in LTBI (*n* = 4), cART naive (*n* = 8), cART (*n* = 4), and cART+3HP (*n* = 6) at preinfection, weeks 3, 9, 11, 13, 15,17, 19, 21, and 23 after *M*. *tuberculosis* infection. Nonparametric Mann-Whitney *U* test was used to compare 2 groups at individual time points. Dotted lines represent cART+3HP treatment (weeks 11–23 after *M*. *tuberculosis* infection). (**C** and **D**) Percentage change in body weight in kg and percentage temperature change in degrees fahrenheit and at preinfection, week 3, week 11, and necropsy in LTBI (*n* = 4), cART naive (*n* = 8), cART (*n* = 4), and cART+3HP (*n* = 6). Nonparametric Mann-Whitney *U* test was used to compare 2 groups at a single time point. **P < 0.01; ***P < 0.001. Data are presented as mean with SD. Made with BioRender.

To report the effects of cART and cART+3HP (3HP = isoniazid+rifapentine) therapy on clinical and pathological outcome in RMs coinfected with *M*. *tuberculosis*/SIV, we utilized our previously published cohorts (11, 12, 23). Specifically, we measured CRP levels, changes in weight, and temperature across the following groups: LTBI (*n* = 4), cART-naive coinfected RMs (*n* = 8), cART-treated RMs (*n* = 4), and cART+3HP-treated RMs (*n* = 6), at predetermined time points (12) (Figure 1A). We reevaluated previously reported comparative clinical and pathological responses in the 4 study groups (11, 12, 23). Development of LTBI was confirmed by culturable bacilli in the bronchoalveolar lavage (BAL) sample (fewer than 1–2 Log_10_CFU) (11, 12), serum C-reactive protein (CRP) levels of ≤ 5 μg/mL and no more than 20% body weight loss or change in body temperature up to week 9 after infection (11, 12, 23). Body weight remained stable overall, with only modest (generally < 5%) and nonsignificant changes from baseline across treatment groups. CRP levels of RMs in the cART+3HP group were significantly lower compared with both cART-naive (*P*<0.0001) and cART-treated RMs (*P*<0.001) (Figure 1B). Furthermore, CRP levels in cART+3HP RMs were not significantly different from LTBI RMs (*P* = 0.44). While the cART naive group exhibited the most change in weight compared with baseline, it was not stastically significantly different from other study groups (Figure 1C). There was no marked change in body temperature (Figure 1D) across all study groups throughout the study period.

We evaluated the effect of cART and cART+3HP on *M*. *tuberculosis* burden by plating the lung tissue (Supplemental Figure 1A; supplemental material available online with this article; https://doi.org/10.1172/jci.insight.199217DS1), lung granulomas (Supplemental Figure 1B), BAL (Supplemental Figure 1C), and spleen (Supplemental Figure 1D) on 7H11 agar plates (14, 15, 17, 20). These data have been reported in our previous publications and are leveraged here to provide clinical context (11, 12, 23). In the cART+3HP group, 5 of 6 RMs had no detectable bacterial load in the lungs at necropsy, compared with only 1 of 4 cART-treated RMs and 2 of 14 cART-naive RMs, with both comparisons yielding statistically significant differences (Supplemental Figure 1A). Notably, the bacterial burdens in the cART+3HP and LTBI (SIV-uninfected) group were comparable (*P* = 0.07), wherein 87.5% and 75% of lung samples were sterile, respectively (Supplemental Figure 1A). Additionally, all 6 RMs in the cART+3HP group had no detectable bacilli in lung granulomas (Supplemental Figure 1B), BAL (Supplemental Figure 1C), or spleen (Supplemental Figure 1D) at necropsy. Furthermore, bacterial burden was significantly lower in the cART+3HP RMs compared with cART-only RMs in both lung tissue (*P* = 0.01) and lung granulomas (*P* = 0.001). In summary, our previously published cohorts show that cART and cART+3HP therapies markedly improve clinical outcomes in RMs coinfected with *M*. *tuberculosis*/SIV. These findings also suggest that cART+3HP is more effective than standard cART therapy in reducing *M*. *tuberculosis* infection and inflammation (11, 23).

### Differential lung pathology in M. tuberculosis/SIV coinfected RMs across treatment regimens.

Pathological evaluation of lung tissue from LTBI, cART-naive, cART-treated, and cART+3HP RMs from our published cohorts (11, 23) revealed marked differences in gross pathology among the groups (Supplemental Figure 2). A low-dose *M*. *tuberculosis* infection resulted in minimal granuloma formation (Supplemental Figure 2A). Additionally, the LTBI group demonstrated markedly reduced pathology, including (a) less pronounced signs of edema, (b) pneumonia, and (c) localized inflammatory foci (Supplemental Figure 2A, indicated by black arrows). In contrast, the cART-naive group displayed distinct pathological features consistent with SIV-induced lung disease, which were more severe than those observed in the LTBI group (Supplemental Figure 2B, indicated by black arrows). These lesions included interstitial pneumonia, marked by inflammation and thickening of the alveolar septa, resulting in impaired pulmonary function. The RMs in the cART-treated group exhibited rare, small granulomas indicating resolution of SIV-driven inflammation (Supplemental Figure 2C, indicated by black arrows). These findings suggest that cART treatment had a stabilizing effect on the disease progression and was effective in controlling the associated pathological lung damage. The RMs in cART+3HP group harbored small, scattered, nonnecrotizing granulomas in the lung lobes (Supplemental Figure 2D, indicated by black arrows). Additionally, rare small aggregates of lymphocytes and macrophages were observed in certain lung sections, suggesting a mild inflammatory response. Overall, the lungs of the cART+3HP-treated RMs exhibited fewer lesions, suggesting that cART+3HP effectively mitigated the severity of pulmonary pathology compared with untreated or less intensively treated groups.

The findings were supported by quantifying the extent of lung involvement across the 4 groups (Supplemental Figure 2E). In the LTBI group, lung involvement was minimal, averaging only 4%–5% of the total lung tissue, consistent with the typically asymptomatic nature of LTBI and effective immune control of bacterial replication. In contrast, lung involvement was significantly greater in the cART-naive group compared with LTBI animals (*P* = 0.03). While cART-treated RMs showed a reduction in lung involvement relative to the cART-naive group, this difference was not statistically significant. Notably, RMs receiving combined cART and 3HP treatment exhibited significantly less lung involvement compared with both the cART-treated (*P* = 0.002) and cART-naive groups (*P* = 0.01). Notably, the combination of cART with 3HP provided the greatest protection, substantially reducing lung involvement and preserving pulmonary architecture to a degree comparable with LTBI alone.

### Impaired IL-21^+^CD4^+^ T cell recovery in lung tissue highlights immune dysregulation during M. tuberculosis/SIV coinfection.

**Figure 2:**
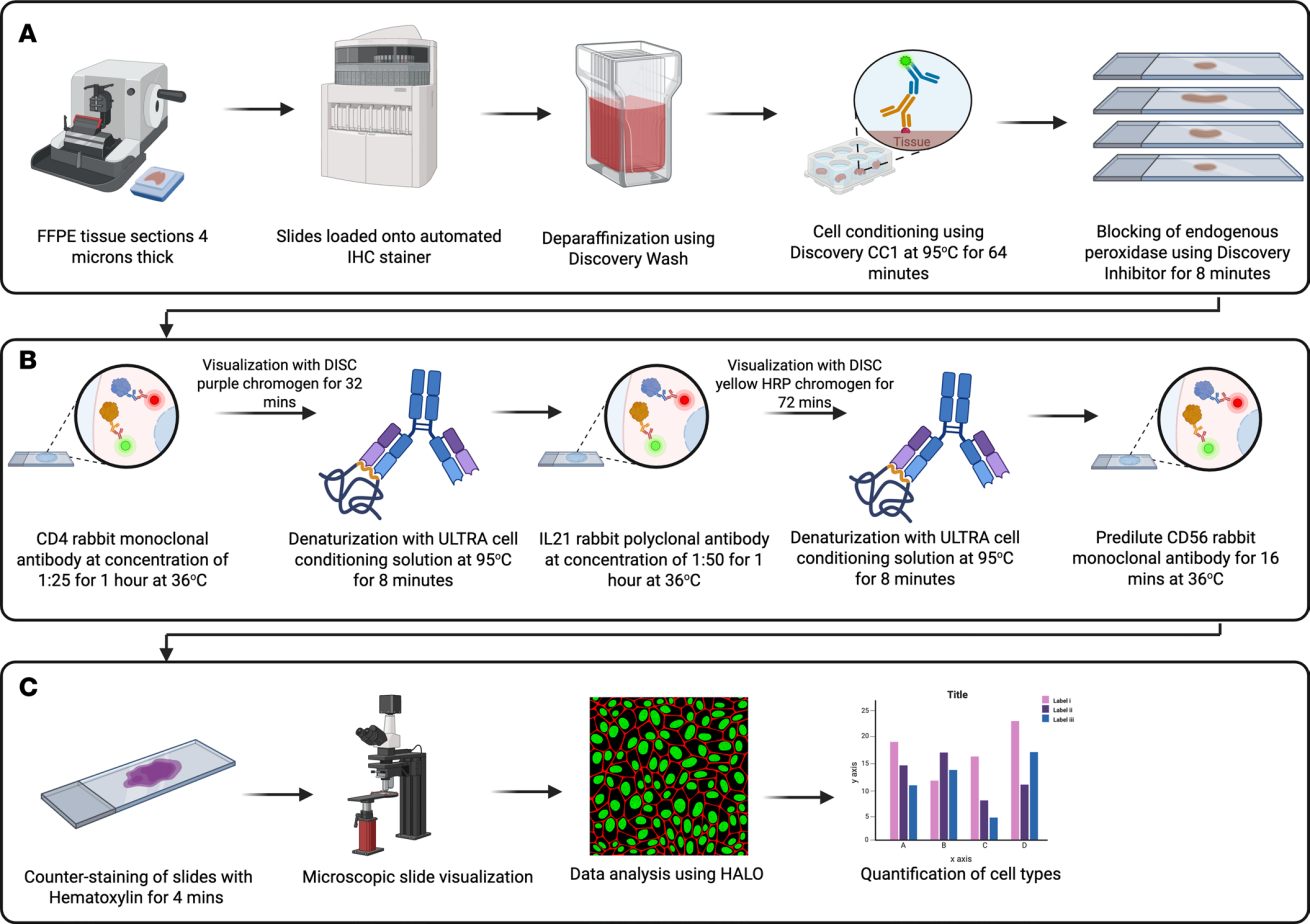
Schematic representing the chromogenic staining workflow. (**A**) Tissue sections were prepared at a standard thickness, mounted on slides, and allowed to dry. Slides were then processed using an automated staining system for the detection of multiple protein markers. The protocol included deparaffinization, antigen retrieval, and blocking of endogenous enzymes. (**B**) Each target protein was detected sequentially. For each marker, the slides were incubated with a primary antibody, followed by detection using a secondary system, and visualized with a chromogenic substrate. Between each marker, a denaturation step was performed to prepare the tissue for subsequent staining. (**C**) After the final staining step, the slides were counterstained to highlight tissue morphology, then prepared for analysis. Made with BioRender.

**Figure 3:**
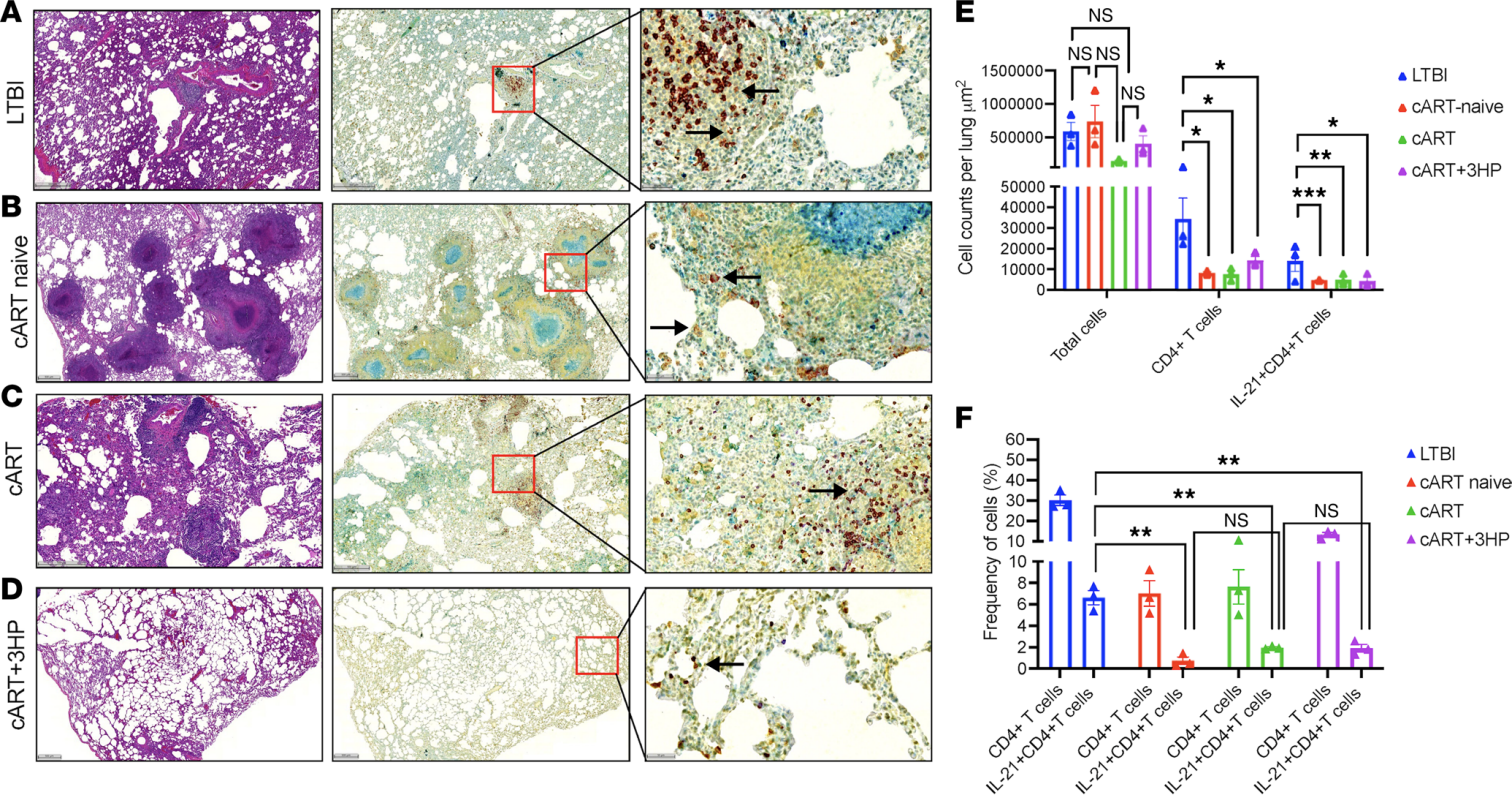
Assessment of IL-21–producing lung CD4^+^ T cells in *M*. *tuberculosis*/SIV coinfected macaques with and without treatment. (**A**–**D**) Formalin-fixed, paraffin-embedded (FFPE) lung sections from rhesus macaques in 4 experimental groups: LTBI (*n* = 3), cART-naive (*n* = 3), cART-treated (*n* = 3), and cART+3HP-treated (*n* = 3) were stained using a multiplex chromogenic protocol. CD4^+^ T cells (red) and IL-21^+^ cells (yellow) were identified, along with double-positive populations: CD4^+^IL-21^+^ cells (orange; indicated by black arrows). Cell quantification was performed using HALO 4.0 image analysis software (Indica Labs), enabling accurate identification and enumeration of single- and double-positive cells. Scale bars, left and center: 500 μm; scale bars, right: 50 μm. (**E**) Cell density (cells/μm² of lung tissue) for total cells, CD4^+^ T cells, and CD4^+^IL-21^+^ T cells. (**F**) Cell frequency (% cells/μm² of lung tissue) for CD4^+^ T cells and CD4^+^IL-21^+^ T cells. Significance was determined using multiple 2-tailed *t* tests using Holm-Sidak method. *P < 0.05; **P < 0.01; ***P < 0.001. Data are presented as mean ± SEM. Made with BioRender.

We used multiplex chromogenic staining with rhesus-specific antibodies according to the workflow provided in Figure 2 to evaluate IL-21 biology in RM FFPE lung sections of LTBI (*n* = 3) (Figure 3A and Supplemental Figure 3), cART naive (*n* = 3) (Figure 3B and Supplemental Figure 4), cART (*n* = 3) (Figure 3C and Supplemental Figure 5), and cART+3HP-treated RMs (*n* = 3) (Figure 3D and Supplemental Figure 6). We used *n* = 3 animals per group, randomly selected from a larger cohort, to evaluate cell counts (Figure 3E) and frequency (Figure 3F) of CD4^+^ T cells producing IL-21. This sample size provides sufficient biological replicates (granulomas) to capture key trends. We propose that each granuloma should be considered an independent biological entity, forming organically and independently of others within the same host. The number of cells positive for either CD4, IL-21, or both CD4 and IL-21 were quantified. While we observe differences in total cell counts between groups, these differences were not statistically significant (Figure 3, A–E). This is likely influenced by the limited sample size (*n* = 3 animals per group) and interanimal variability in cellular responses between study macaques. As shown in the data, individual animals within the same group can exhibit variable total cellularity, which reduces statistical power. To further contextualize these findings, we compared total cell counts from each experimental group to baseline (normal lung tissue) (Supplemental Figure 7A). This comparison demonstrates that, relative to baseline lung tissue, there is evidence of increased cellularity across disease groups, even though differences between experimental groups at necropsy are not statistically significant. Notably, while anti-TB treatment does not restore CD4^+^IL-21^+^ T cell frequencies in lung tissue of *M*. *tuberculosis*/SIV coinfected animals to levels observed in LTBI, systemic IL-21 levels are reconstituted to levels comparable to LTBI following treatment (Supplemental Figure 7B). Together, these findings indicate that IL-21 dysregulation in *M*. *tuberculosis*/SIV coinfection is predominantly lung specific and persists locally despite systemic immune reconstitution.

A marked difference in CD4^+^ T cell counts was observed in the lungs across the 4 groups. Compared with the LTBI group (Figure 3, A and E), RMs in the cART-naive (Figure 3B) (*P* = 0.03), cART (Figure 3C) (*P* = 0.04), and cART+3HP groups (*P* = 0.04) (Figure 3D) exhibited significantly lower numbers of CD4^+^ T cells. These findings are consistent with our previous flow cytometry data, which shows that SIV coinfection in LTBI RMs leads to substantial depletion of CD4^+^ T cells in both BAL and lung tissues (12). Notably, treatment with cART alone or in combination with 3HP resulted in partial restoration of these cells, though levels remained below those seen in LTBI animals (12, 23). The significant reduction of IL-21^+^CD4^+^ T cells in the cART-naive (Figure 3B) (*P* = 0.002), cART (Figure 3C) (*P* = 0.03), and cART+3HP groups (*P* = 0.03) (Figure 3D) versus the LTBI group (Figure 3, A and E) aligns with reports of lower IL-21 in TB patients (25) and SIV-infected RMs (22), highlighting the potential role of IL-21 in *M*. *tuberculosis* and SIV immunity and disease progression. Animals with LTBI exhibit readily detectable frequencies of IL-21^+^CD4^+^ T cells in the lung (approximately ~6%–7% of CD4^+^ T cells). Following SIV coinfection, the frequency of IL-21^+^CD4^+^ T cells is markedly reduced (to ~0.2%–0.5%), representing a disproportionate decline relative to the reduction in total CD4^+^ T cell frequencies (Figure 3F). Notably, despite partial recovery of overall CD4^+^ T cells with cART, IL-21^+^CD4^+^ T cell frequencies remain low and are not restored to LTBI levels, indicating selective and persistent impairment of this subset. We normalized IL-21 expression to CD4^+^ T cells by calculating the IL-21^+^CD4^+^/CD4^+^ ratio for each animal. Using this approach, LTBI animals exhibited IL-21/CD4 ratios of 0.20–0.38 (mean, 0.28), whereas cART-naive animals showed markedly reduced ratios of 0.05–0.16 (mean, 0.10), indicating impaired IL-21 expression on a per-CD4^+^ cell basis. Animals receiving cART alone exhibited intermediate ratios (0.12–0.19; mean, 0.14) that remained below LTBI levels while cART+3HP animals demonstrated partial restoration of the IL-21/CD4 ratio (0.15–0.28; mean, 0.23). Notably, despite partial or complete systemic reconstitution of IL-21/CD4 ratios following treatment, lung CD4^+^IL-21^+^ T cell frequencies were not restored to LTBI levels. Together, these data indicate that IL-21 dysregulation in *M*. *tuberculosis*/SIV coinfection is not solely attributable to numerical CD4^+^ T cell loss but reflects a qualitative, lung-localized defect in IL-21 expression that persists despite systemic immune reconstitution.

To determine whether this effect reflects global CD4^+^ T cell dysfunction or subset-specific dysregulation, we examined additional CD4^+^ effector populations (Supplemental Figure 8). IFN-γ^+^CD4^+^ T cell responses increased from ~0%–0.5% preinfection to ~1.5%–3% during LTBI and SIV infection, with partial maintenance or recovery following cART. Similarly, TNF-α^+^CD4^+^ T cells were present at ~1.5%–3.5% during LTBI and SIV coinfection and, although reduced with cART, remained detectable (~0.5%–1.5%). IL-17^+^CD4^+^ T cells showed more modest changes, with frequencies generally remaining below ~1% across groups. In contrast, IL-21–producing CD4^+^ T cells showed a greater magnitude of loss and failed to recover with cART, despite relative preservation of other effector CD4^+^ T cell subsets. To further determine whether pulmonary CD4^+^ T cells exhibit characteristics consistent with tissue retention, we quantified expression of markers CD103 (Supplemental Figure 9, A–D and I) and CD69 (Supplemental Figure 9, E–H and J). Across groups, a distinct subset (~20%–35%) coexpressed CD103 (Supplemental Figure 9, A–D and I). A proportion of lung CD4^+^ T cells expressed CD69 (Supplemental Figure 9, E–H and J), comprising approximately 55%–70% of total CD4^+^ T cells, with the highest frequencies observed in LTBI animals (Supplemental Figure 9, E and J). Although overall CD4^+^ T cell numbers were reduced in *M*. *tuberculosis*/SIV-coinfected macaques (Supplemental Figure 9, I and J), the relative frequency of CD4^+^CD69^+^ and CD4^+^CD103^+^ T cells within the lung parenchyma and granulomatous regions remains detectable. Notably, cART or cART+3HP did not change the proportion of CD103^+^ T cells (Supplemental Figure 9I) but partially restored the CD69^+^ subset compared with untreated *M*. *tuberculosis*/SIV animals (Supplemental Figure 9J).

A linear regression analysis was conducted to evaluate the relationship between the frequency of IL-21^+^CD4^+^ T cells and lung bacterial burden (Supplemental Figure 10A) and percentage lung pathology (Supplemental Figure 10B) in cART naive macaques. The variable, Log_10_ IL-21^+^CD4^+^ T cells, was found to be a critical predictor of lung burden. The regression model was statistically significant, *P* = 0.025, indicating that variation in IL-21^+^CD4^+^ T cell levels explains variability in lung bacterial load. To determine whether the observed association was specific to IL-21^+^CD4^+^ T cells or simply reflected global CD4^+^ T cell depletion, we performed additional linear regression analyses examining total CD4^+^ T cell frequencies in relation to lung bacterial burden and lung pathology. Notably, total CD4^+^ T cell counts did not correlate with either lung bacterial burden (Supplemental Figure 11A) or percent lung pathology (Supplemental Figure 11B). These data support the interpretation that IL-21 expression represents a qualitative functional deficit rather than simply a quantitative reduction in CD4^+^ T cell numbers. Future studies with larger cohorts and broader subset-specific quantification will allow more comprehensive comparative analyses. Overall, treatment of *M*. *tuberculosis*/SIV coinfected RMs with cART alone or concurrent cART+3HP treatment, is insufficient in restoring IL-21 levels in the lung.

### Differential IL-21 expression by CD4^+^ T cells in granulomatous versus nongranulomatous lung tissue.

We further investigated IL-21 production by CD4^+^ T cells in granuloma-associated regions of lung tissue of *M*. *tuberculosis*/SIV coinfected, cART naive RMs (KG40, KR44, JF47) compared with nongranulomatous areas (Supplemental Figure 12, A and B). While the role of Th1-associated cytokines in granuloma formation, especially in LTBI, is well documented (25, 26), the contribution of the Th17 cytokine, IL-21, to granuloma dynamics in TB and TB/HIV coinfection remains unclear. Our use of a NHP model, combined with multiplex chromogenic staining and HALO quantification, provides an ideal framework for examining IL-21 production in lungs of *M*. *tuberculosis*/SIV coinfected RMs. A higher percentage of CD4^+^IL-21^+^ T cells (except in KG40) (Supplemental Figure 12C) associated with nongranulomatous regions in all *M*. *tuberculosis*/SIV coinfected RMs. Analysis of individual animals in the cART-naive group demonstrates that CD4^+^IL-21^+^ T cells are present in both granulomatous and nongranulomatous lung regions, indicating that these cells can access granulomatous tissue (Supplemental Figure 12C). However, there is a consistent intraanimal trend toward higher frequencies of CD4^+^IL-21^+^ T cells in nongranulomatous regions. This pattern suggests that, while IL-21–producing CD4^+^ T cells are not excluded from granulomas, they may preferentially be retained in less organized inflammatory environments. Nongranulomatous regions may provide a microenvironment more permissive for sustained IL-21 expression, whereas granulomas characterized by dense macrophage cores, hypoxia, and high local inflammatory pressure may limit either the differentiation or persistence of IL-21–producing CD4^+^ T cells. In Supplemental Figure 12D, this trend reaches statistical significance in select treatment groups but not others, likely reflecting lesion heterogeneity and interanimal variability. Accordingly, we interpret these data as supporting a model in which CD4^+^IL-21^+^ T cells contribute to immune regulation in the lung primarily outside mature granulomas, while still being capable of entering granulomatous regions.

### Recovery of colonic CD4^+^ and IL-21^+^CD4^+^ T cells following cART or cART+3HP in M. tuberculosis/SIV coinfection.

HIV infection causes a marked depletion of both the absolute number and percentage of CD4^+^ T cells, particularly in the gut, during the acute phase of infection. Limited access to human colon tissue samples has created substantial gaps in our understanding of CD4^+^ T cell dynamics during *M*. *tuberculosis*/HIV coinfection. Evaluating IL-21^+^CD4^+^ T cell recovery in the colon of RMs complements our lung findings by determining whether treatment restores this functionally important subset across distinct mucosal sites.

**Figure 4:**
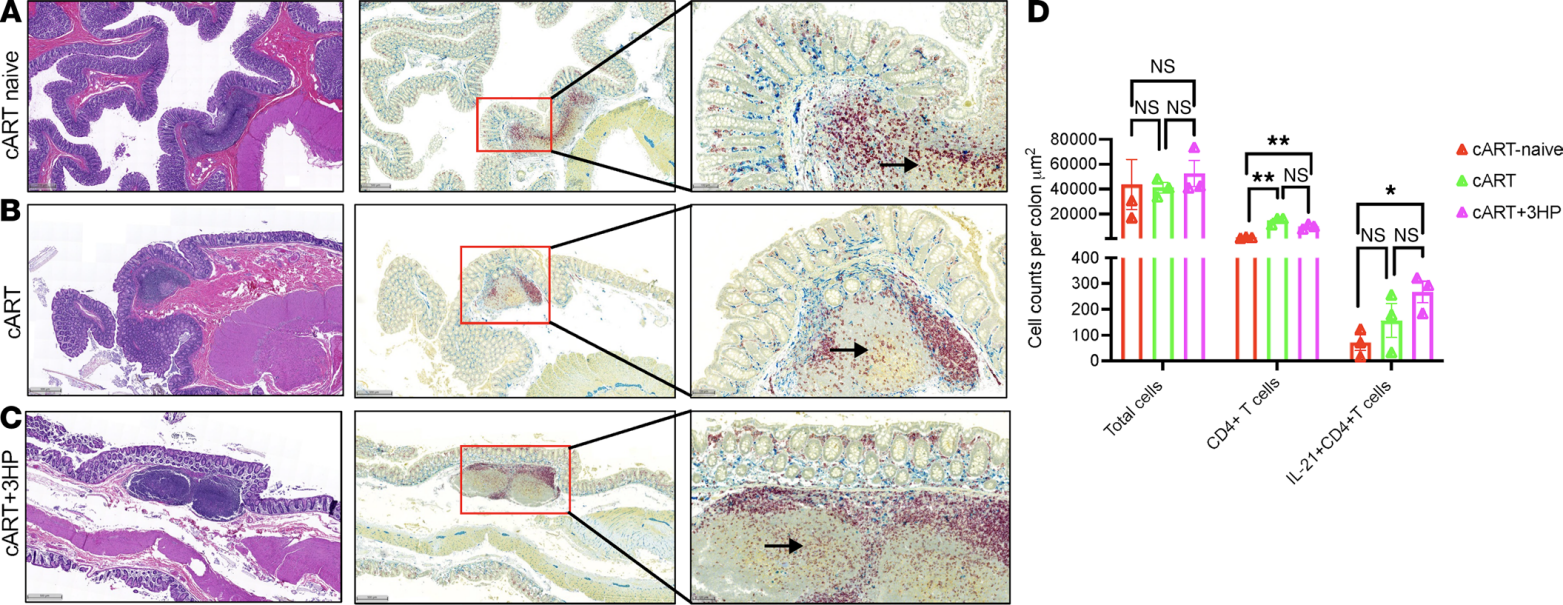
Chromogenic staining and quantification of CD4^+^ T cells and CD4^+^IL-21^+^ T cells in colon tissue. (**A**–**C**) Representative images of colon sections from cART naive (*n* = 3), cART (*n* = 3), and cART+3HP (*n* = 3) stained for CD4^+^ T cells and dual-positive CD4^+^IL-21^+^ T cells (marked with black arrows) using chromogenic IHC. (**D**) Quantification of total cells, CD4^+^ T cells, and CD4^+^IL-21^+^ T cells, represented as the number of positive cells per μm² of colon tissue. Data are presented as mean ± SEM. Significance was determined using multiple 2-tailed *t* tests using Holm-Sidak method. *P < 0.05; **P < 0.01. Data are presented as mean ± SEM. Made with BioRender. Scale bars, left and center: 500 μm; scale bars, right: 100 μm.

To this end, we quantified the total number of CD4^+^ T and CD4^+^ IL-21^+^ T cells in the colon sections from 3 groups of *M*. *tuberculosis*/SIV-coinfected RMs using chromogenic staining: cART naive (Figure 4A), cART (Figure 4B), and cART+3HP treated (Figure 4C). The primary objective was to evaluate the effect of treatment on reconstitution of total CD4^+^ T cells and their subset producing IL-21, compared with untreated cART naive RMs. There was no significant difference in total cells in the 3 study groups (cART naive, cART-treated, and cART+3HP-treated) (Figure 4D). Expectedly, cART-treated (*P* = 0.001) and cART+3HP-treated (*P* = 0.001) RMs showed significantly higher total CD4^+^ T cell count in the colon compared with cART naive RMs (Figure 4D). Both treatment regimens reconstituted CD4^+^ T cells to similar levels with no significant difference between these 2 groups (*P* = 0.07). A significantly higher number of CD4^+^ IL-21^+^ T cells was seen in cART+3HP-treated RMs compared with cART naive RMs (*P* = 0.02) (Figure 4D). Overall, initiating cART or cART+3HP early in coinfection enabled reconstitution of CD4^+^ T cells and their subset, producing IL-21 in colon compared with untreated RMs. However, whether this reconstitution is long -term, functional, and metabolically active remains to be studied.

### Mapping the spatial immune landscape of lung tissue in M. tuberculosis/SIV coinfection.

**Figure 5:**
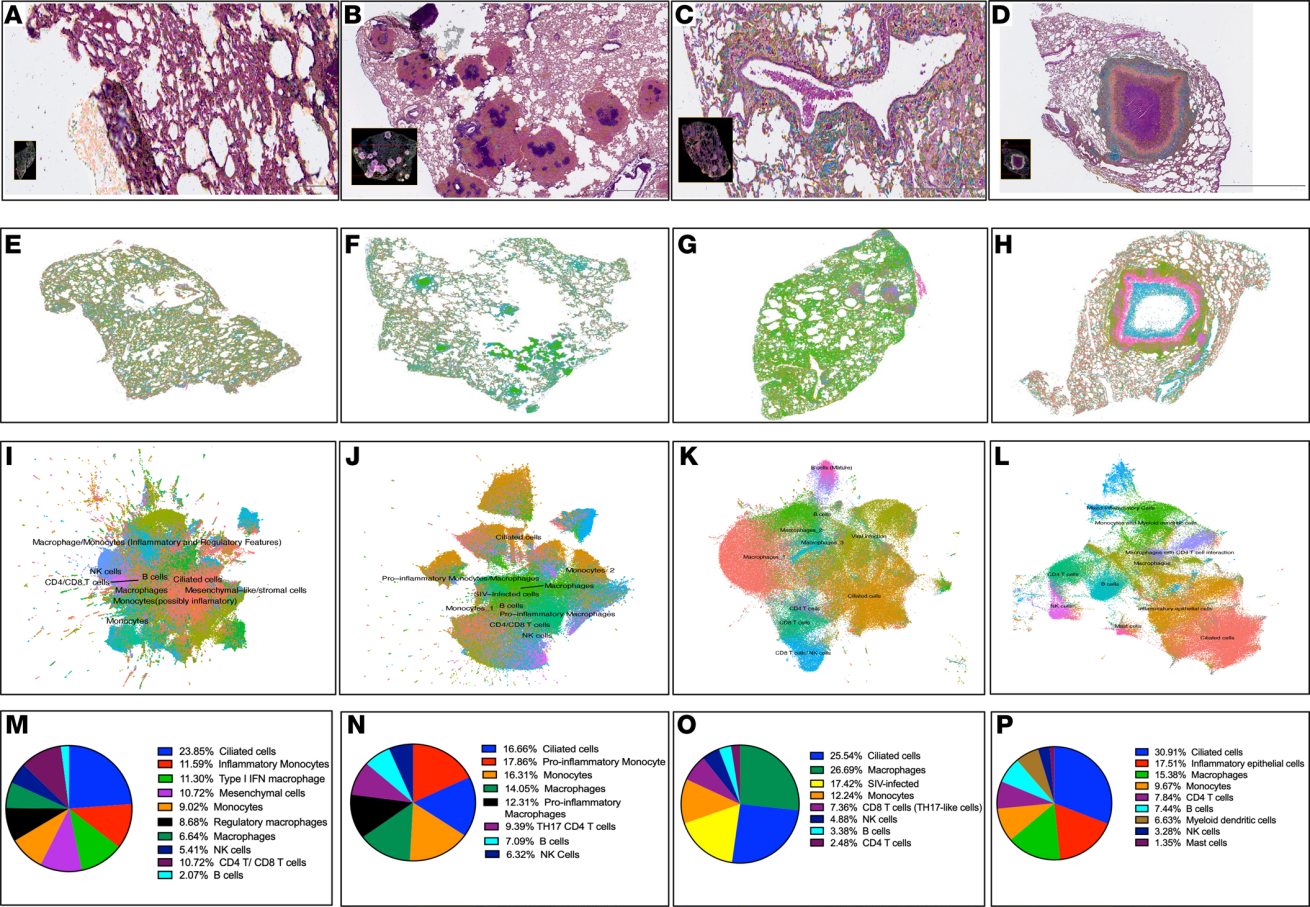
Spatial transcriptomic profiling of lung tissue from RMs across LTBI and HIV treatment conditions using Xenium. Xenium transcriptomics was performed on lung tissue sections from RM from (LTBI), cART-naive, cART-treated, and cART+3HP-treated groups. (**A**–**D**) Representative region of lung tissue from RM in LTBI (*n* = 1, 2 regions) (**A**), cART naive (*n* = 1, 2 regions) (**B**), cART (*n* = 1, 3 regions) (**C**), and cART+3HP (*n* = 1, 4 regions) (**D**) showing H&E staining overlaid with spatial transcriptomic data acquired using the Xenium platform. H&E staining provides morphological context, with nuclei stained in blue/purple (hematoxylin) and cytoplasm/extracellular matrix in pink (eosin). Xenium signals represent the spatial distribution of custom panel genes, shown as colored spots overlaid on the histological image. Scale bar: 2,000 μm. (**E**–**H**) Dim plots showing transcriptional profiles of ROIs derived from spatial transcriptomic analysis of lung tissues in LTBI (*n* = 1, 2 regions) (**E**), cART-naive (*n* = 1, 2 regions) (**F**), cART (*n* = 1, 3 regions) (**G**), and cART+3HP (*n* = 1, 4 regions) (**H**) RM. Each point represents a spatially defined ROI, with color coding indicating group identity or unsupervised clustering based on transcriptomic similarity. Plots illustrate distinct transcriptional landscapes associated with disease state and treatment regimen. (**I**–**L**) Uniform Manifold Approximation and Projection (UMAP) plot displaying transcriptionally distinct cell clusters derived from spatial transcriptomic profiling of lung tissue from LTBI (*n* = 1, 2 regions) (**I**), cART-naive (*n* = 1, 2 regions) (**J**), cART (*n* = 1, 3 regions) (**K**), and cART+3HP (*n* = 1, 4 regions) (**L**) RM. Each point represents an individual cell or transcriptomic feature, colored by cluster identity. Clusters were identified through unsupervised clustering and annotated based on expression of canonical marker genes. (**M**–**P**)Parts-of-whole graph illustrating the relative abundance of transcriptionally defined cell clusters identified in lung tissue from LTBI (*n* = 1, 2 regions) (**M**), cART-naive (*n* = 1, 2 regions) (**N**), cART (*n* = 1, 3 regions) (**O**), and cART+3HP (*n* = 1, 4 regions) (**P**) RM using Xenium analysis. Each segment represents a distinct cell cluster, with size corresponding to the number of cells within that cluster. Made with BioRender.

We examined *IL-21*, *CD4*, *CD56*, Th1-, and Th17-associated gene expression patterns within the context of tissue architecture. To this end, we performed Xenium spatial transcriptomics (Supplemental Figure 13) on lung tissue section from RM in LTBI, cART naive, cART-treated, and cART+3HP-treated groups (Supplemental Table 1). We selected the region for analysis in Xenium based on quality control metrics provided in the Xenium Analysis Summary, ensuring optimal data integrity and signal quality (Supplemental Figure 14). The overlaid H&E and Xenium images from RM in LTBI (Figure 5A), cART naive (Figure 5B), cART (Figure 5C), and cART+3HP (Figure 5D) were used to visually identify and annotate key anatomical and pathological features within the lung tissue. Granulomas were identified as dense, rounded immune cell aggregates with defined borders, often corresponding to transcriptionally active clusters in Xenium. Necrotic zones within granulomas appeared as central pale or acellular regions in H&E, often surrounded by a dense rim of immune cells, suggesting immune-mediated tissue damage; these zones often showed reduced transcript density in Xenium. Immune cell-rich areas were characterized by dense nuclei in H&E and high expression of immune markers in Xenium, typically forming clusters suggestive of localized immune responses.

Xenium spatial transcriptomic data from all regions were imported into Seurat for visualization and downstream analysis using unsupervised clustering. K-means clustering results (K = 10) from the original Xenium output were added to the Seurat object as metadata. After preprocessing using Seurat SCTransform workflow, to determine cell type identities, cluster-specific marker genes were extracted using the FindAllMarkers function with the following parameters (min.pct = 0.1, logfc.threshold = 0.1). The resulting marker gene lists were compared against known reference markers (Supplemental Table 2) to assign cell type annotations for each cluster. A custom 176-plex Xenium gene panel exhibited high sensitivity and specificity for *IL-21*, *CD4*, *NCAM1*, Th1-, and Th17-associated gene expression patterns. The granuloma-associated regions were chosen in the LTBI (Figure 5E), cART naive (Figure 5F), cART (Figure 5G), and cART+3HP (Figure 5H) to map gene expression. Uniform Manifold Approximation and Projection (UMAP) clustering was performed to identify and visualize cell clusters in regions of interest (ROI) in the lung tissue section from RM in LTBI (Figure 5I), cART naive (Figure 5J), cART (Figure 5K), and cART+3HP (Figure 5L) groups based on their gene expression. Overall, the common cell subsets detected in each ROI include monocytes, macrophages, CD4^+^ T cells, CD8^+^ T cells, B cells, and NK cells (Figure 5, M–P). In addition to the expected subsets, ROI from lung tissue section of the LTBI RM exhibited inflammatory monocytes and Type I IFN-responsive macrophages (Figure 5M). Coinfection with SIV led to a pronounced presence of Th17 CD4^+^ T cells and proinflammatory macrophages (Figure 5N). Treatment with cART elevated the Th17-like response in CD8^+^ T cells but exhibited continued expression of markers associated with viral infection (*S100A9*, *IL1R1*, *TMPRSS2*, *ICAM1*) (Figure 5O). cART+3HP-treated RMs showed presence of mast cells and myeloid dendritic cells that were not seen in other groups pointing to a potential role of inflammatory response to enable bacterial clearance (Figure 5P). In our spatial transcriptomics analysis (Figure 5O), inflammatory monocytes were not detected above threshold levels in lung sections from animals receiving cART. We interpret this finding as biologically meaningful and consistent with reduced pulmonary inflammation following effective antiviral therapy. Importantly, spatial transcriptomics detects cell populations based on their transcriptional signatures within the sampled regions; therefore, the absence of a detectable inflammatory monocyte cluster likely reflects their low abundance or absence in these treated lungs.

**Figure 6:**
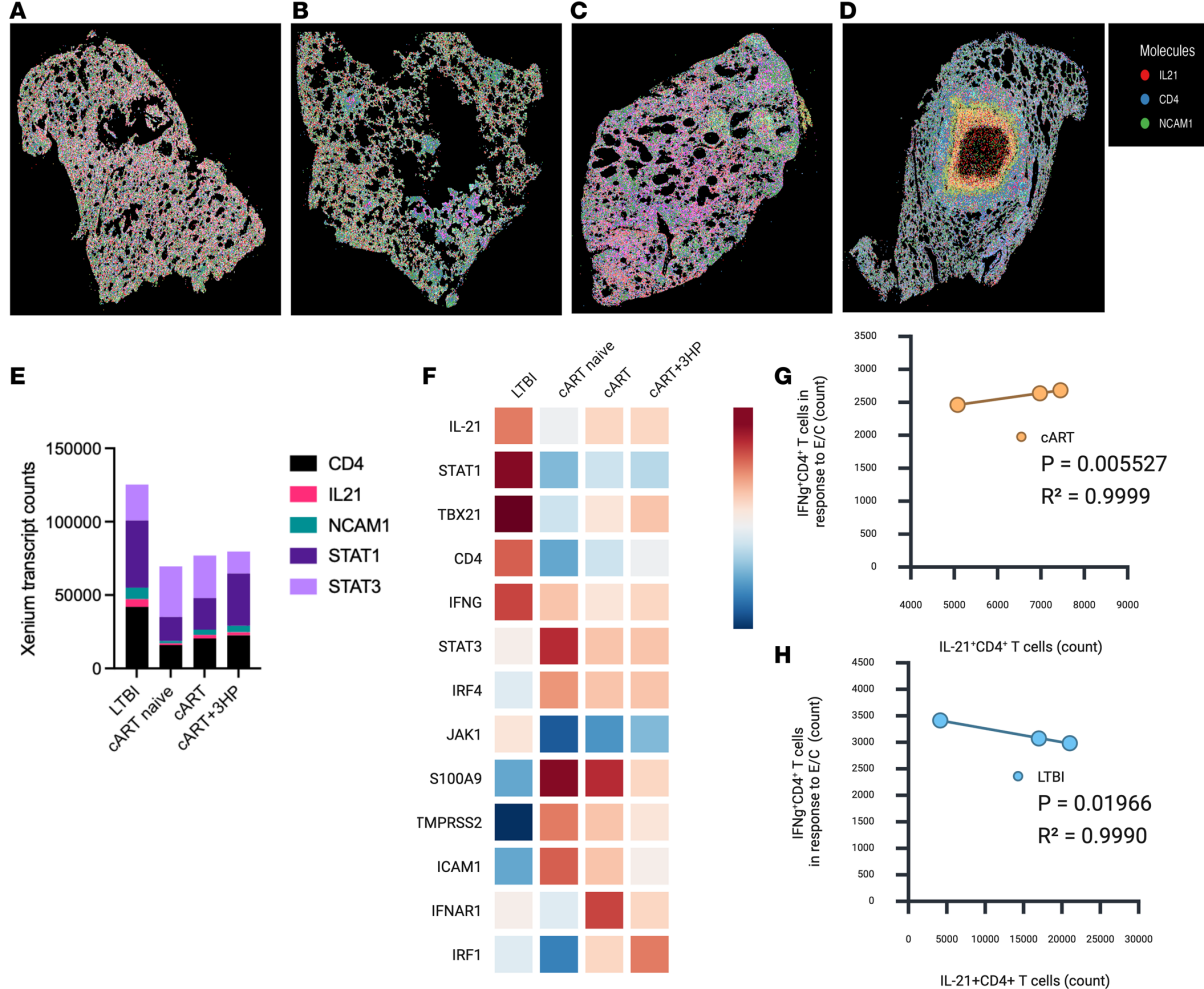
Quantification of Th1 and Th17 response associated transcript counts in lung tissue. (**A**–**D**) Representative dimensionality reduction (dim) plots from Xenium spatial transcriptomics show the spatial distribution and expression intensity of *CD4*, *IL21*, and *NCAM1* transcripts within selected ROIs from LTBI (*n* = 1, 2 regions) (**A**), cART naive (*n* = 1, 2 regions) (**B**), cART (*n* = 1, 3 regions) (**C**), and cART+3HP (*n* = 1, 4 regions) (**D**) RM. Each point represents a detected transcript, with color intensity reflecting relative abundance. (**E**) Stacked bar plots show the total transcript counts for *CD4*, *IL21*, *NCAM1*, *STAT1*, and *STAT3* within defined regions of interest (ROIs) in lung tissue sections from RMs across 4 experimental groups. Data were obtained from Xenium spatial transcriptomics analysis, with transcript abundance reflecting localized gene expression in the lung microenvironment. Comparisons across groups highlight changes in immune cell presence (*CD4*, *NCAM1*), cytokine signaling (*IL21*), and transcriptional regulation (*STAT1*, *STAT3*) in response to *M*. *tuberculosis*/SIV infection and treatment. (**F**) Log_2_ fold change of Th1 associated genes (*IL-21*, *STAT1*, *TBX21*, *CD4*, *IFNG*), Th17 associated genes (*STAT3*, *IRF4*, *JAK1*), viral infection associated genes (*S100A9*, *TMPRSS2*, *ICAM1*), and IFN response associated genes (*IFNAR1*, *IRF1*) in LTBI (*n* = 1, 2 regions), cART naive (*n* = 1, 2 regions), cART (*n* = 1, 3 regions), and cART+3HP (*n* = 1, 4 regions) RM. (**G** and **H**) Linear regression analysis of IL-21^+^CD4^+^ T cell count vs IFNγ^+^CD4^+^ T cell count in response to ESAT-6/CFP-10 in lung tissue (*n* = 3) in cART-treated RMs (**G**) and in LTBI RMs (*n* = 3) (**H**). Statistical significance was assessed by calculating the *P* value for the slope coefficient in a linear regression model, with significance defined as *P* < 0.05, using R version 4.2.2. Made with BioRender.

We quantified transcript counts of *CD4*, *IL-21*, *NCAM1*, *STAT1* (Th1-associated), and *STAT3* (Th17-associated) within the same ROI in lung tissue sections used for cluster annotation. In lung tissue from the LTBI RM, *CD4*, *IL-21*, and *NCAM1* transcripts displayed a uniform spatial distribution (Figure 6, A and E), accompanied by a balanced expression of *STAT1* and *STAT3* and moderate transcript counts of *CD4*, *IL-21*, and *NCAM1*, suggesting a mixed Th1/Th17 immune profile. In contrast, the cART-naive RM exhibited elevated *STAT3* transcript counts and reduced *CD4*, *IL-21*, *NCAM1*, and *STAT1* counts compared with LTBI (Figure 6, B and E), consistent with previous cluster analyses (Figure 5N). The cART-treated RM showed reduced transcript counts of *CD4*, *STAT1*, *IL-21*, and *NCAM1* compared with LTBI, though the spatial distribution remained similar (Figure 6, C and E). Notably, the cART+3HP group demonstrated the lowest *STAT3* transcript counts among all groups (Figure 6, D and E), while *STAT1* levels were comparable with LTBI. However, despite concurrent viral and bacterial treatment, Th1-associated transcripts in cART+3HP remained lower than in LTBI (Figure 6, D and E). Overall, these findings suggest an imbalanced Th17/Th1 response in cART-naive macaques, characterized by elevated *STAT3* and diminished Th1-associated gene expression, a dysregulation not fully corrected by cART alone. While cART+3HP appears more effective in mitigating Th17 dominance and partially restoring immune balance, Th1 transcript counts, particularly IL-21, remain lower than those in LTBI animals, indicating persistent IL-21 dysregulation in the lung microenvironment despite treatment.

Xenium-based analysis of differential gene expression in CD4^+^ T cell clusters from lung tissues of RM from LTBI, cART-naive, cART-treated, and cART+3HP-treated groups revealed distinct transcriptional profiles associated with disease and treatment status (Figure 6F). LTBI RM exhibited higher expression of *IL21* and Th1-associated genes, including *STAT1*, *TBX21*, *CD4*, and *IFNG*, compared with all SIV-infected groups (Figure 6F). In contrast, cART-naive RM showed elevated expression of Th17-associated genes (*STAT3* and *IRF4*) relative to LTBI, cART, and cART+3HP groups (Figure 6F). Notably, *STAT3* transcripts in cART-naive animals were predominantly localized around granulomatous regions, suggesting spatial enrichment of Th17-type responses in areas of active inflammation. Furthermore, cART-naive RM exhibited higher expression of genes associated with viral infection and inflammation, including *S100A9*, *TMPRSS2*, and *ICAM1*, compared with cART+3HP-treated animals (Figure 6F). Elevated expression of these viral-associated genes was also observed in cART-treated RMs (Figure 6F), indicating persistence of viral transcriptional activity in the lung despite reduced viral titers (12). To assess whether IL-21^+^CD4^+^ T cells contribute to *M*. *tuberculosis*–specific effector responses, a linear regression analysis was performed to examine whether the frequency of IL-21^+^CD4^+^ T cells in the lungs predicts the frequency of *M*. *tuberculosis*–specific IFN-γ^+^CD4^+^ T cells in cART-treated RM (Figure 6G), LTBI RM (Figure 6H), cART naive (Supplemental Figure 15A), and cART+3HP-treated RM (Supplemental Figure 15B), following ex vivo stimulation of lung cells with ESAT-6/CFP-10 (E/C). The analysis revealed that IL-21^+^CD4^+^ T cell counts were a predictor of IFN-γ^+^CD4^+^ T cell responses in LTBI (*P* = 0.02) and cART-treated RM (*P* = 0.006). This indicates a statistically significant linear relationship between the 2 cell populations, suggesting that IL-21^+^CD4^+^ T cells may play a key role in supporting or correlating with *M*. *tuberculosis*–specific IFN-γ responses in the lung during antiretroviral therapy. There was no significant relationship between frequency of IL-21^+^CD4^+^ T cells in the lungs and *M*. *tuberculosis*–specific IFN-γ^+^CD4^+^ T cells in cART naive (*P* = 0.43) or cART+3HP-treated RM (*P* = 0.52). Together, these results demonstrate that, in *M*. *tuberculosis*+SIV coinfected animals that were cART-naive or treated with cART+3HP, there was no linear association between IL-21^+^CD4^+^ T cells and *M*. *tuberculosis*–specific IFN-γ^+^CD4^+^ T cells, indicating that IL-21–producing CD4^+^ T cells do not quantitatively track with Th1 effector function in these settings.

## Discussion

This study investigated the modulation of IL-21 in the context of LTBI and *M*. *tuberculosis*/SIV coinfection using a biologically relevant animal model, specifically within the pulmonary compartment. Despite extensive research into the role of IL-21 in SIV infection (22, 27), there remains a critical gap in understanding the IL-21 modulation during *M*. *tuberculosis* infection, particularly in NHP models. *M*. *tuberculosis* stimulation of peripheral blood cells from healthy individuals with LTBI revealed that CD4^+^ and NK T cells are the primary sources of IL-21. In contrast, CD4^+^ T cells isolated from individuals with active TB produced lower levels of IL-21 in response to *M*. *tuberculosis* (28). These findings are consistent with our observations in the lungs of LTBI macaques, which similarly exhibited robust IL-21 production. In contrast, *M*. *tuberculosis*/SIV coinfected macaques that did not receive cART or cART+3HP treatment exhibited reduced IL-21 levels. Notably, this decline was independent of a lower frequency of CD4^+^ T cells, reduced CD4 transcript levels, and diminished expression of NCAM1 transcripts in the cART-naive group.

Prior studies have shown that IL-21 supports the survival and proliferation of CD4^+^ T cells and augments IFN-γ production, all of which contribute to the containment of *M*. *tuberculosis* infection (20, 29). Furthermore, IL-21 has been implicated in sustaining Tfh cell responses and facilitating the development of effective memory responses, key components of long-term immune control in LTBI (30, 31). Notably, treatment with cART, either alone or in conjunction with 3HP, resulted in a partial restoration of CD4^+^ T cells and CD4^+^IL-21^+^ T cells. This finding aligns with previous animal studies demonstrating that, while cART can improve overall CD4^+^ T cell counts, the restoration of specific functional subsets, such as IL-21–producing Th cells, is often incomplete (32). Similarly, human studies have shown that, even with effective cART, immune recovery to mycobacterial antigens remains suboptimal (33). Our recent work supports this, revealing that SIV infection compromises the immune response and that cotreatment with cART and 3HP, while beneficial, does not fully normalize functional CD4^+^ T cell levels (23).

The gastrointestinal (GI) tract, including the colon, is a major site of CD4^+^ T cell depletion during both HIV and SIV infections, with profound immunologic damage that is only partially reversed with cART (34, 35). Studies have shown that this compartment retains some capacity for immune reconstitution following cART (36). In our study, treatment with cART or cART+3HP led to only marginal increases in CD4^+^ and CD4^+^IL-21^+^ T cell counts in the colon compared with cART-naive *M*. *tuberculosis*/SIV coinfected RMs. This limited reconstitution may reflect early initiation of therapy during the acute phase of viral infection, which is known to result in more effective control of viral replication and bacterial burden (12, 37, 38). However, it remains unclear whether these reconstituted CD4^+^ and CD4^+^IL-21^+^ T cells in the colon are fully functional and capable of long-term persistence.

The imbalanced Th17-Th1 immune response in cART-naive macaques, marked by elevated *STAT3* expression and diminished *CD4*, *IL-21*, *STAT1*, and *NCAM1*, supports earlier observations that chronic HIV/SIV infection alters Th17-Th1 balance while impairing Th1-mediated control of *M*. *tuberculosis* (39, 40). *STAT3*-driven responses, while protective in some contexts, can promote persistent inflammation and tissue damage, potentially exacerbating *M*. *tuberculosis* pathology in the absence of effective bacterial clearance mechanisms (41, 42). The limited restoration of *IL-21* and *CD4* transcript levels under cART treatment is consistent with studies showing that cART, although capable of improving CD4^+^ T cell counts, does not fully reconstitute the functional capacity of pathogen-specific T cells (43). Our data suggest that this incomplete immune recovery may be particularly pronounced in tissue compartments like the lung, where local immune suppression and fibrosis may create microenvironments resistant to reconstitution. Moreover, the persistence of viral response genes (*ICAM1*, *S100A9*, *IL1R1*, *TMPRSS2*) even after cART further implies residual immune activation and dysregulation at tissue level, a phenomenon observed in PLHIV, often associated with microbial translocation and systemic inflammation (44, 45).

Spatial transcriptomic analysis provided additional insight into the organization of immune responses within the lung; however, these data should be interpreted with caution given the limited sampling, with one representative animal per group, and therefore are best considered exploratory and hypothesis-generating. Within these constraints, we observed differences in Th1- and Th17-associated transcripts, including *STAT1* and *STAT3*, across disease and treatment conditions, with *M*. *tuberculosis*/SIV coinfection associated with increased *STAT3* expression and reduced Th1-associated transcripts, partially modulated by treatment. These observations are correlative and do not establish causality. Similarly, the relationship between IL-21 expression and *STAT1/STAT3* signaling remains inferential, as no functional experiments were performed to directly assess the impact of IL-21 on macrophage activity or downstream signaling pathways in this model. Accordingly, we limit our interpretation to associations between IL-21 expression and transcriptional patterns, and further studies incorporating functional approaches will be required to define the mechanistic role of IL-21 in pulmonary immune responses during *M*. *tuberculosis*/SIV coinfection.

IL-21 is critical for maintaining effective helper functions, including support of CD8^+^ T cells, and balanced IFN responses. Its persistent loss after SIV coinfection despite viral suppression and TB therapy may impair immune regulation within granulomas, favoring chronic IFN signaling and macrophage dysfunction rather than coordinated antimicrobial immunity. Together, these data support a model in which SIV coinfection of LTBI drives Th17-skewed, STAT3-associated inflammatory programming while selectively depleting IL-21–producing CD4^+^ T cells. Subsequent treatment reduces inflammatory amplification but is associated with persistent Type I IFN signatures, marked by IRF1 and IFNAR1 expression, that are linked to pathological immune responses in *M*. *tuberculosis* infection.

Importantly, the addition of 3HP to cART appears to modify the immune landscape more favorably, reducing *STAT3* expression while restoring *STAT1* and enhancing the presence of myeloid dendritic cells and mast cells, cell types associated with improved pathogen sensing, antigen presentation, and early granuloma formation (46, 47). These shifts may reflect a therapeutic synergy in which the antimicrobial effects of isoniazid and rifapentine reduce *M*. *tuberculosis* burden, alleviating chronic immune stimulation and allowing for a more regulated immune reconstitution. Our analysis of IL-21^+^CD4^+^ T cell distribution within granulomatous and nongranulomatous regions suggests a trend toward higher frequencies outside granulomas; however, this pattern was not consistent across all animals and did not reach statistical significance in all comparisons. These findings likely reflect lesion heterogeneity and interanimal variability. Based on the persistent deficiencies in IL-21–producing CD4^+^ T cells observed in pulmonary compartment despite cART and 3HP treatment, IL-21 emerges as a compelling candidate for HDT in *M*. *tuberculosis*/SIV coinfection. Supplementing cART and cART+3HP with exogenous IL-21 could address these deficits by reinforcing Th1 responses via *STAT1*, reinstating balanced Th1/17 functionality, and promoting NK cell–mediated cytotoxicity, thereby enhancing both systemic and local immunity.

## Methods

### Sex as a biological variable.

Our study examined male and female animals, and similar findings are reported for both sexes.

### M. tuberculosis and SIV coinfection of RMs.

This study did not enroll any new RMs but rather used lung tissue sections from 22 RMs of completed studies (11, 12, 23) (Supplemental Table 3). Briefly, 12 RMs from a SPF colony were included from studies completed at TNBRC (7, 11) and 10 RMs from a specific pathogen–free colony from studies completed at SNPRC (12, 23). All RMs were exposed to approximately 10 CFU *M*. *tuberculosis* CDC1551 (BEI Resources, catalog NR13649) via aerosol (7, 48, 49). Nine weeks later, 18 macaques were coinfected with 300_TCID50_ of SIV_mac239_ via i.v. (11, 12) (SIV was provided by the Tulane National Biomedical Research Center, Covington, USA). The remaining 4 macaques served as LTBI-only controls (Group 1). SIV infection was confirmed by qPCR measuring plasma viral loads. Upon confirmation, the 18 coinfected macaques were divided into 3 groups: Group 2 (8 RMs) served as coinfected controls (cART naive), Group 3 (4 RMs) with cART 2 weeks after SIV infection, and Group 4 (6 RMs) with cART+3HP 2 weeks after SIV infection. All the RMs were euthanized by week 24, which was the predetermined study endpoint, or at earlier time points if RMs became clinically unwell in accordance with humane endpoint criteria.

### cART and 3HP administration to RMs.

The cART regimen for the coinfected NHPs included a combination of 20 mg/kg of (R)-9-(2-phosphonylmethoxypropyl) adenine (PMPA, tenofovir, Gilead Sciences), 30 mg/kg of 2′,3′-dideoxy-5-fluoro-3′-thiacytidine (FTC, emtricitabine, Gilead Sciences), and 2.5 mg/mL of the integrase inhibitor dolutegravir (DTG, ViiV Healthcare). These drugs were administered daily through s.c. injection as a cocktail mixed with the vehicle KLEPTOSE (Roquette, parenteral grade 346111) at previously established dosages (11, 12). The coinfected RMs in group 4 were also given a weekly oral dose of 15 mg/kg isoniazid and 15 mg/kg rifapentine for 12 weeks, starting at week 12 after aerosol infection and continuing through week 23 after TB infection. Veterinary staff closely monitored oral intake to ensure proper consumption of the medications (23, 38).

### Measurement of bacterial burden in RMs.

The bacterial burden was assessed throughout the study period as previously outlined (11, 12). Viable *M*. *tuberculosis* burden was evaluated at necropsy in samples from BAL, lungs, spleen, and individual granulomas from all 4 study groups. The tissues were homogenized, and the homogenate was then serially diluted and plated on Middlebrook 7H11 agar, which supported the growth of *M*. *tuberculosis*. After an incubation period of 4–5 weeks, colony-forming units (CFUs) were counted, providing a quantifiable measure of the bacterial load within the sampled tissue.

### Pathological examination of H&E-stained tissue sections.

Following collection, the lung tissues were fixed in 10% neutral buffered formalin and embedded in paraffin for histological examination. Sections were cut at 5 μm thickness and stained with H&E. To assess the extent of lung involvement, stereology scores were generated, with a board-certified veterinary pathologist evaluating the percentage of lung tissue affected by pathology. HALO scores served as an indication for the true percentage of lung affected. The lesions in each lung lobe were scored for pleural thickening, intralobular septae inflammation, perivasculitis, pneumocyte hyperplasia, and lymphadenitis.

### Multiplex chromogenic staining.

Tissue sections for IHC were cut at 4 μm thick, mounted onto positively charged slides, and allowed to air dry overnight. Slides were then loaded onto the Discovery Ultra IHC/ISH automated stainer for the detection of the CD4, and IL-21 protein markers. Deparaffinization occurred using Discovery Wash (Roche, catalog 950-510). Cell conditioning was performed using Discovery CC1 (Roche, catalog 950-500) at 95°C for 64 minutes. The blocking of endogenous peroxidase occurred using Discovery Inhibitor (Roche, catalog 760-4840) for 8 minutes. Slides were then incubated with CD4 (EP204) rabbit monoclonal antibody (Cell Signaling, catalog 48274) at a concentration of 1:25 for 1 hour at 36°C. Detection was performed by using Anti-rabbit HQ (Roche, catalog 760-4815) for 8 minutes followed by Anti-HQ HRP (Roche, catalog 760-4820) for 8 minutes, both at 36°C. CD4 (EP204) was visualized by applying DISC Purple chromogen (Roche, catalog 760-229) for 32 minutes. Slides were then denatured using ULTRA Cell Conditioning Solution (ULTRA CC2, Roche, catalog 950-223) at 95°C for 8 minutes. Next, slides were incubated with IL-21 rabbit polyclonal antibody (Abcam, catalog 154767) at a concentration of 1:50 for 1 hour at 36°C. Detection was performed by using Anti-rabbit HQ for 8 minutes followed by Anti-HQ HRP for 8 minutes, both at 36°C. Visualization of IL-21 was achieved by applying DISC Yellow HRP chromogen (Roche, catalog 760-250) for 72 minutes. The slides were counterstained using Hematoxylin (Roche, catalog 760-2021) for 4 minutes followed by Bluing Reagent (Roche, catalog 760-2037) for 4 minutes.

### HALO quantification.

The multiplex chromogenic IHC stained lung and colon sections were digitized by scanning at 40× magnification in the Zeiss Axio Scan Z1 whole slide imager (Zeiss, Germany), and the whole slide digital images were analyzed using HALO 4.0 software (Indica Labs, NM, USA). The multiplex IHC analysis module v3.4.9 was optimized by a board-certified pathologist to identify and quantify the specific cell population including CD4^+^ T cells, CD4^+^ T cells producing IL-21, and other IL-21–producing cells. The area of organized granuloma and nonorganized granulomatous inflammation areas were annotated by a board-certified pathologist. Nuclei were identified based on hematoxylin staining and was used to quantify total number of cells in the tissue area examined (Supplemental Table 4).

### FFPE tissue sectioning, placement, and quality assessment for xenium in situ analysis.

Formalin-fixed paraffin-embedded (FFPE) tissue blocks were sectioned at a nominal thickness of 5 μm using a rotary microtome equipped with a low-profile disposable blade. Sections were floated briefly on an RNase-free 40°C–42°C water bath to flatten. Tissue sections were then placed onto Xenium *In Situ v1* slides. Slides were air-dried at room temperature (RT) for 5 minutes, incubated at 40°C in the oven for 3 hours to hours to promote tissue adhesion. Following quality assessment, FFPE tissue sections were subjected to the Xenium *In Situ v1* pretreatment workflow to enable RNA target accessibility. Hybridized padlock probes underwent probe ligation step to ensure a unique level of probe specificity to the target region. Ligated probes underwent rolling cycle amplification to enhance 586 signal intensity by producing hundreds of copies of the gene-specific barcode. Xenium slides containing FFPE tissue sections were then loaded for imaging and analysis on the xenium analyzer instrument for high-throughput, automated in situ analysis.

### Xenium and H&E integration.

To overlay H&E and Xenium images, the original*.svs* H&E image files were converted to.*ome.tiff* format using QuPath v0.5.1. These files were then imported into Xenium Explorer v3.2, where they could be directly overlaid with the corresponding spatial transcriptomics data. This enabled visual alignment of histological features with spatial gene expression, allowing for integrated analysis of tissue architecture and molecular signatures. The resulting overlaid images were exported and saved as.png files for documentation and downstream analysis.

### Unsupervised clustering and cell-type annotation.

Xenium spatial transcriptomic data from all regions were imported into Seurat for visualization and downstream analysis. K-means clustering results from the original Xenium output were added to the Seurat object as metadata. After preprocessing using Seurat SCTransform workflow, to determine cell type identities, cluster-specific marker genes were extracted using the FindAllMarkers() function with the following parameters (min.pct = 0.1, logfc.threshold = 0.1). The resulting marker gene lists were compared against known reference markers (Supplemental Table 2) to assign cell type annotations for each cluster. Cluster identification was performed using unbiased automated softwares, Azimuth_ALV1L1, Azimuth_ALV1L2, Azimuth_ALV2L4, and Clustermole. The genes were matched to expression clusters using the Human Protein Atlas (https://www.proteinatlas.org/about) and Gene Cards (The Human Genome Database; https://www.genecards.org). We further confirmed the cluster annotation using the list of published signature gene list (23). Heatmaps of top marker gene expression by cluster is shown for LTBI (Supplemental Figure 16), cART naive (Supplemental Figure 17), cART (Supplemental Figure 18), and cART+3HP (Supplemental Figure 19). Xenium morphology image quality control for RMs from LTBI, cART naive, cART, and cART+3HP is provided in Supplemental Figure 20. Xenium data were generated using the 10x Genomics Xenium in situ platform, and this analysis is based on Xenium Analyzer outputs provided by Tulane Center for Aging Spatial Multi-Omics Core.

### Statistics.

Statistical analyses were performed using GraphPad Prism (version 10.0) and R (version 4.2.2). Data are presented as mean ± SD for clinical and bacterial burden measurements and mean ± SEM for histologic and immunologic quantifications, as indicated in the figure legends. For comparisons between 2 groups at individual time points, nonparametric 2-tailed Mann-Whitney *U* tests were used due to small sample sizes. For multiplex chromogenic IHC quantifications, multiple 2-tailed *t* tests with Holm-Šidák correction were applied to control for multiple comparisons. Spatial transcriptomic analyses from Xenium datasets were processed using Seurat, and gene expression differences were summarized as log_2_ fold changes across groups. Correlations between immunologic parameters and functional or disease outcomes were assessed using linear regression or Pearson correlation analysis, with significance determined from the slope coefficient. Statistical significance was defined as *P* < 0.05.

### Study approval.

All infected animals were housed under Animal Biosafety Level 3 facilities at the Southwest National Primate Research Center, where they were treated according to the standards recommended by AAALAC International and the *Guide for the Care and Use of Laboratory Animals* (National Academies Press, 2011). The study procedures were approved by the IACUC of the Texas Biomedical Research Institute.

### Data availability.

All data necessary for the evaluation of the conclusions in the article are presented in the article and/or in the supplemental materials and methods. Values for all data points in graphs are reported in the Supporting Data Values file.

## Author contributions

RS contributed conceptualization, data curation, formal analysis, funding acquisition, methodology, project administration, writing of the original draft, review, and editing. VS contributed chromogenic staining, H&E analysis, HALO software analysis, and manuscript review. RDE and CC contributed chromogenic staining, H&E staining, and methodology. SPS contributed manuscript review and editing. AAM and JQB performed Xenium staining, visualization, and slide processing. CX and SK contributed Xenium tranacriptomic analysis. EJD performed pathology analysis. SM contributed manuscript review. MP contributed project consultation, manuscript writing, review, and editing.

## Conflict of interest

The authors have declared that no conflict of interest exists.

## Funding support

This work is the result of NIH funding, in whole or in part, and is subject to the NIH Public Access Policy. Through acceptance of this federal funding, the NIH has been given a right to make the work publicly available in PubMed Central.

- NIH investigator-awards 1R56AI184089-01A1, 1R21AI170148-01, 1K01OD031898-01 to RS.
- NIH investigator-awards R01AI181710, UM1AI164562, ERASE HIV grant NIAID/NHLBI/NIDDK/NIMH/NINDS/NIDA 1UM1AI16456 to MP.
- P51 OD011133, S10OD028732, and S10OD032443 P30AI168439 (Texas D-CFAR, member RS, and P30AI161943 (IN-TRAC; Members: RS and SPS).
- NIGMS COBRE grants P20GM103629 and P30GM145498 to SMJ.

## Supplementary Material

Supplemental data

Supporting data values

## Version 1 05/05/2026

In-Press Preview

## Version 2 06/22/2026

Electronic publication
