# Supplementary material for: Modulation of pulmonary IL-21 expression during latent TB and M. tuberculosis/SIV coinfection
Source: JCI Insight. 2026 May 5;11(12):e199217. doi: 10.1172/jci.insight.199217 (PMC13313536; doi:10.1172/jci.insight.199217)
Supplement: Supplemental data [file jciinsight-11-199217-s059.pdf]

Supplemental Figure 1. Bacterial burden ($\log_{10}$CFU/g or $\log_{10}$CFU/mL) was determined in (**A**) lung, (**B**) lung granulomas, (**C**) BAL, (**D**) spleen at necropsy in LTBI (*n*=4), cART naive (*n*=8), cART (*n*=4), and cART+3HP (*n*=6) by homogenizing the tissues and plating on agar plates. Nonparametric Mann-Whitney test was used to compare 2 groups. *P < 0.05; **P < 0.01; ***P < 0.001; ****P < 0.0001. Data are presented as mean with SD.

Supplemental Figure 2. Hematoxylin & Eosin-stained lung tissue to study the cellular and granulomatous pathology in (**A**) LTBI (*n*=3), (**B**) cART naive (*n*=3), (**C**) cART (*n*=3), and (D) cART+3HP (*n*=3). Scale bars: 5 mm. (**E**) Percentage of lung involvement was calculated by a board-certified pathologist by quantification of the number of lesions per lobe in LTBI (*n*=3), cART naive (*n*=3), cART (*n*=3), and cART+3HP (*n*=3). Nonparametric Mann-Whitney test was used to compare 2 groups. *P < 0.05; **P < 0.01; ***P < 0.001; ****P < 0.0001. Data are presented as mean with SD.

Supplemental Figure 3. Chromogenic staining to detect CD4 and IL-21 in lung tissues from RMs infected with low dose *Mtb* infection (*n*=3). Representative image of lung section from (**A,B**) GP50, (**C,D**) HV02 and (**E,F**) JF47. Scale bars: 5000 μm, 2000 μm. $CD4^+$ T cells (red) and $IL\text{-}21^+$ cells (yellow) were identified, along with double-positive populations: $CD4^+IL\text{-}21^+$ cells (orange; indicated by black arrows).

Supplemental Figure 4. Chromogenic staining to detect CD4 and IL-21 in lung tissues from RMs co-infected with *Mtb*/SIV (*n*=3). Representative image of lung section from (**A,B**) JH07, (**C,D**) KG40 and (**E,F**) KR44. Scale bars: 5000 μm, 2000 μm. $CD4^+$ T cells (red) and $IL\text{-}21^+$ cells (yellow) were identified, along with double-positive populations: $CD4^+IL\text{-}21^+$ cells (orange; indicated by black arrows).

Supplemental Figure 5. Chromogenic staining to detect CD4 and IL-21 in lung tissues from RMs co-infected with *Mtb*/SIV and treated with cART (*n*=3). Representative image of lung section from (**A,B**) 33343, (**C,D**) 33994 and (**E,F**) 34741. Scale bars: 1 cm, 5000 μm, 2000 μm. $CD4^+$ T cells (red) and $IL\text{-}21^+$ cells (yellow) were identified, along with double-positive populations: $CD4^+IL\text{-}21^+$ cells (orange; indicated by black arrows).

Supplemental Figure 6. Chromogenic staining to detect CD4 and IL-21 in lung tissues from RMs co-infected with *Mtb*/SIV and treated with cART+3HP (*n*=3). Representative image of lung section from (**A,B**) 41876, (**C,D**) 41882 and (**E,F**) 41901. Scale bars: 5000 μm, 2000 μm. $CD4^+$ T cells (red) and $IL\text{-}21^+$ cells (yellow) were identified, along with double-positive populations: $CD4^+IL\text{-}21^+$ cells (orange; indicated by black arrows).

Supplemental Figure 7. (**A**) Total lung cell counts (cells per lung, μm^2) measured in uninfected (baseline) (*n*=3),

LTBI (*n*=3), cART-naïve (*n*=3), cART (*n*=3), and cART+3HP (*n*=3) groups. Each dot represents an individual subject; horizontal lines with error bars indicate mean ± SEM. No statistically significant differences were observed between groups (ns, not significant). (**B**) Plasma IL-21 concentrations (pg/mL) in the same groups. Each dot represents an individual subject; horizontal lines with error bars indicate mean ± SEM. Significant differences between indicated groups are denoted by brackets and asterisks (**P* < 0.05); ns indicates not significant. Nonparametric Mann-Whitney test was used to compare 2 groups.

Supplemental Figure 8. Bar graphs show the percentage of IL-21$^+$ CD4$^+$ T cells relative to IFN-γ^+, IL-17$^+$, and TNFα^+ CD4$^+$ T cell subsets in LTBI (*n*=3), cART-naïve (*n*=3), cART (*n*=3), and cART+3HP (*n*=3) groups. Black bars represent IL-21$^+$ CD4$^+$ T cells, pink bars represent IFN-γ^+ CD4$^+$ T cells, teal bars represent IL-17$^+$ CD4$^+$ T cells, and purple bars represent TNFα^+ CD4$^+$ T cells. Individual symbols denote single subjects, and bars represent mean ± SEM. Statistical significance between indicated groups is shown by brackets with asterisks (**P*< 0.05, ***P* < 0.01); ns indicates not significant. Nonparametric Mann-Whitney test was used to compare 2 groups.

Supplemental Figure 9. (**A-D**) Representative immunohistochemistry images showing CD4$^+$CD103$^+$ cells in lung tissue from (**A**) LTBI (*n*=3), (**B**) cART-naive (*n*=3), (**C**) cART-treated (*n*=3), and (**D**) cART+3HP-treated (*n*=3) animals. Red arrows indicate CD4$^+$CD103$^+$ T cells. (E-H) Representative images of CD4$^+$CD69$^+$ T cells. Representative IHC images showing CD4$^+$CD9$^+$ T cells in lung tissue from (**E**) LTBI (*n*=3), (**F**) cART-naive (*n*=3), (**G**) cART-treated (*n*=3), and (**H**) cART+3HP-treated (*n*=3) animals. Red arrows indicate CD4$^+$CD69$^+$ T cells. Scale bars; 500 μm, 100 μm. (**I**) Quantification of total CD4$^+$ T cells and CD4$^+$CD103$^+$ T cells per lung area (μm²). (**J**) Quantification of total CD4$^+$ T cells and CD4$^+$CD69$^+$ T cells per lung area (μm²). Data are shown as mean ± SEM; **P* < 0.05; ns, not significant. Nonparametric Mann-Whitney test was used to compare 2 groups.

Supplemental Figure 10. Linear regression analysis of (**A**) Log10 IL-21$^+$CD4$^+$ T cells vs Log10 CFU/g lung burden (*n*=3) and (**B**) Log10 IL-21$^+$CD4$^+$ T cell vs % lung pathology (*n*=3). Statistical significance was assessed by calculating the *p*-value for the slope coefficient in a linear regression model, with significance defined as $P < 0.05$, using R version 4.2.2.

Supplemental Figure 11. (**A**) Correlation between $\log_{10}$ CD4$^+$ T cell counts and lung *Mtb* burden in cART-naive and cART-treated animals. Pearson correlation analysis showed no significant association in either group (cART-naive: r = −0.3906, *P* = 0.3723; cART: r = 0.8261, *P* = 0.1906). (**B**) Correlation between $\log_{10}$ CD4$^+$ T cell counts and lung pathology scores in cART-naive and cART-treated animals. No significant correlations were observed (cART-naive: r = 0.1203, *P* = 0.4616; cART: r = 0.9048, *P* = 0.14). Each symbol represents an individual animal. Statistical significance was defined as α = 0.05 (ns, not significant).

Supplemental Figure 12. Representative chromogenic images from cART naïve RMs, (**A**) KG40 and (**B**) KR44

demonstrating the granulomatous and non-granulomatous areas of analysis. Scale bars: 5000 μm. Stacked bar graphs showing (**C**) %CD4$^+$IL-21$^+$T cells in granulomatous and non-granulomatous areas in the lung tissue section of cART naïve RMs, JF47, KG40 and KR44. (**D**) %CD4$^+$IL-21$^+$ T cells in cells in granulomatous and non-granulomatous areas in the lung tissue section of cART naïve (*n*=3), cART (*n*=3) and cART+3HP (*n*=3) groups. Data are shown as mean ± SEM; *$P < 0.05$; ns, not significant. Nonparametric Mann-Whitney test was used to compare 2 groups.

Supplemental Figure 13. Schematic illustrating the four main steps of the Xenium platform used for spatial gene expression analysis. (**A**) Tissue analysis: Formalin-fixed tissue sections were mounted and prepared for spatial profiling, preserving tissue morphology and RNA integrity. (**B**) cDNA synthesis: In situ reverse transcription was performed, converting captured mRNA molecules into spatially barcoded complementary DNA (cDNA) while maintaining their native spatial context. (**C**) Gene expression detection: Targeted probes hybridized to the cDNA, enabling multiplexed detection of selected transcripts with subcellular resolution. (**D**) Data analysis: Spatial gene expression data were processed to generate high-resolution transcript maps, identify cell types, and quantify gene expression patterns across defined tissue regions.

Supplemental Figure 14. The figure displays gene-specific metrics such as transcript detection counts, spatial localization accuracy, and signal-to-background ratio for key genes of interest in region of interest in lung tissue sections from RMs in (**A**) LTBI, (**B**) cART-naïve, (**C**) cART and (**D**) cART+3HP treated groups. Total number of transcripts detected per gene across analyzed lung tissue sections from RMs in (**E**) LTBI, (**F**) cART-naïve, (**G**) cART and (**H**) cART+3HP treated groups using the Xenium platform. Transcript counts were derived from decoding analysis, which identifies and assigns individual RNA molecules to target genes based on probe hybridization patterns. This analysis reflects gene-specific expression abundance across all regions of interest. (**I**) Table displaying key decoding performance metrics across lung tissue sections analyzed using the Xenium platform. These metrics provide an overview of decoding efficiency, tissue integrity, and transcript localization quality across samples and support robust spatial gene expression analysis.

Supplemental Figure 15. (**A**) Pearson correlation between total IL-21$^+$CD4$^+$ T cell counts and IFNγ$^+$CD4$^+$ T cells responding to *Mtb* cell wall (CW) antigen in cART-naive animals. No significant association was observed ($P = 0.4321$, $R^2 = 0.6058$). (**B**) Pearson correlation between IL-21$^+$CD4$^+$ T cell counts and *Mtb* CW-specific IFN-γ$^+$CD4$^+$ T cell responses in cART+3HP-treated animals. No significant association was detected ($P = 0.5237$, $R^2 = 0.4628$). Each symbol represents an individual animal. Statistical significance was defined as $P < 0.05$.

Supplemental Figure 16. Heatmap of top marker gene expression by cluster in region on interest in LTBI RM. The table provides cluster numbers, top markers and annotation.

Supplemental Figure 17. Heatmap of top marker gene expression by cluster in region on interest in cART naive

RM. The table provides cluster numbers, top markers and annotation.

Supplemental Figure 18. Heatmap of top marker gene expression by cluster in region on interest in cART RM. The table provides cluster numbers, top markers and annotation.

Supplemental Figure 19. Heatmap of top marker gene expression by cluster in region on interest in cART+3HP RM. The table provides cluster numbers, top markers and annotation.

Supplemental Figure 20. Xenium morphology image QC gallery for RMs in (**A**) LTBI, (**B**) cART naïve, (**C**) cART and (**D**) cART+3HP. Image QC provides image quality based on cell segmentation. Xenium system generates morphology images, including DAPI (nuclei stain), ATP1A1/CD45/E-Cadherin for cell boundary, 18s for interior RNA and alphaSMA/Vimentin for interior protein. X axis represents the X -coordinate of a point in the image (μm) and Y axis represents the fluorescence intensity of the stained morphology images (photon equivalent - pe).

Supplemental Table 1. List of samples and experimental design information for Xenium platform. This project focuses on dissecting TB/SIV co-Infection and treatment response in macaque lung tissues. It involves a total of 4 slides, each representing an animal from one of the following groups.

Supplemental Table 2. List of reference markers used for annotation of cell clusters. After preprocessing using Seurat SCTransform workflow, to determine cell type identities, cluster-specific marker genes were extracted using the FindAllMarkers function. The resulting marker gene lists were compared against known reference markers to assign cell type annotations for each cluster.

Supplemental Table 3. Demographics of the study animals including age, sex, therapy initiation, and Mamu type of study macaques.

Supplemental Table 4. Total lung tissue area (μm^2) quantified for each animal in the LTBI, cART-naive, cART-treated, and cART+3HP-treated groups. Values represent the cumulative area analyzed per animal.

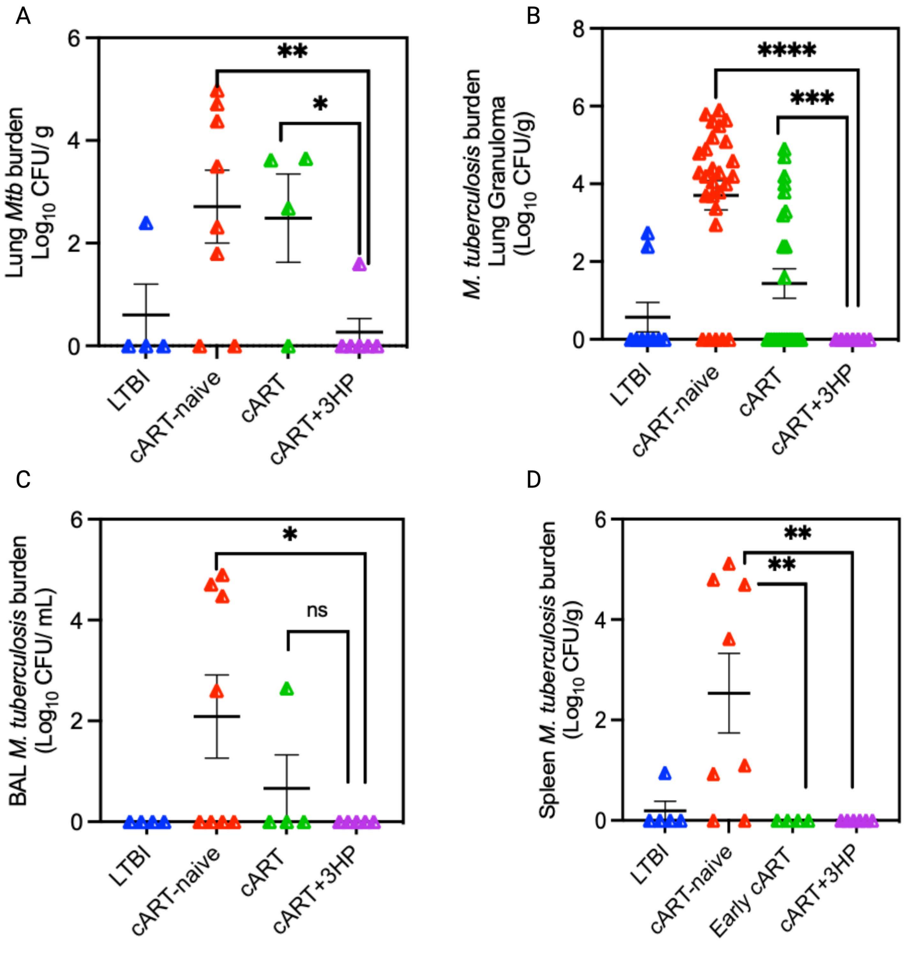

A
Lung Mtb burden Log10 CFU/ g
**
*
LTBI
cART-naive
cART
cART+3HP
B
M. tuberculosis burden Lung Granuloma (Log10 CFU/g)
****
***
LTBI
cART-naive
cART
cART+3HP
C
BAL M. tuberculosis burden (Log10 CFU/ mL)
*
ns
LTBI
cART-naive
cART
cART+3HP
D
Spleen M. tuberculosis burden (Log10 CFU/g)
**
**
LTBI
cART-naive
Early cART
cART+3HP

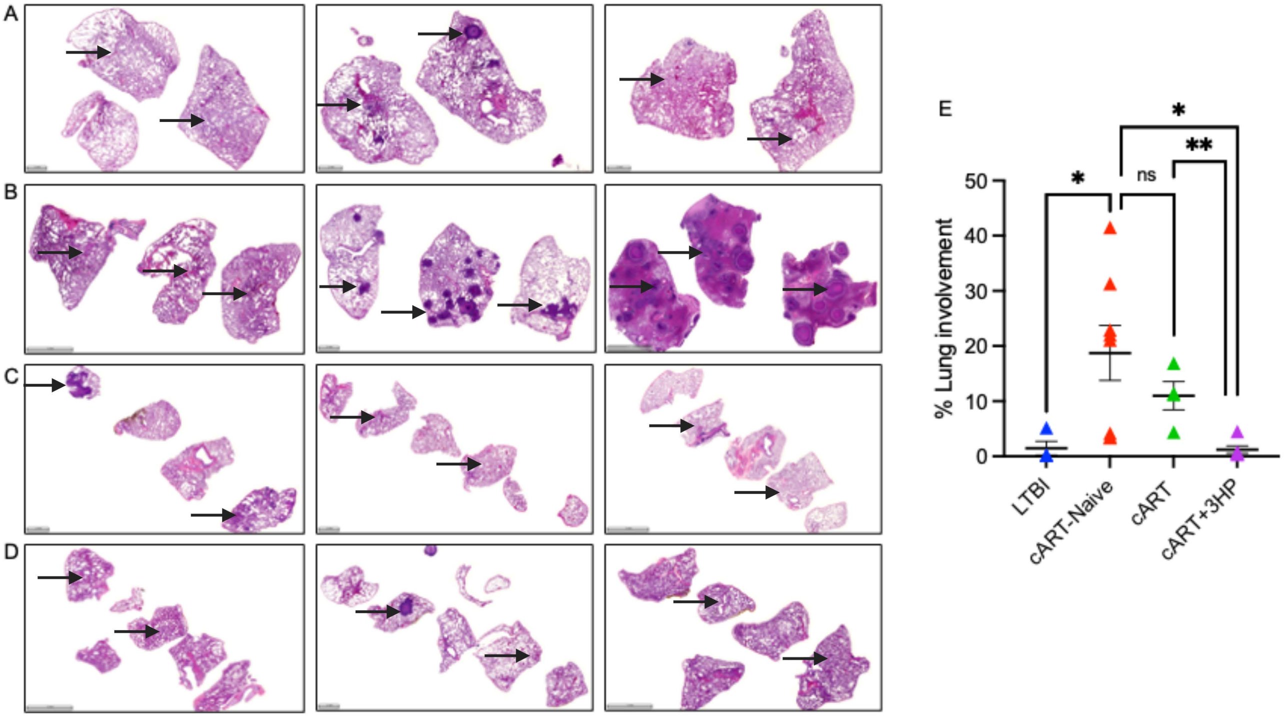
A
B
C
D
E
% Lung involvement
0
10
20
30
40
50
*
ns
*
**
LTBI
cART-Naive
cART
cART+3HP

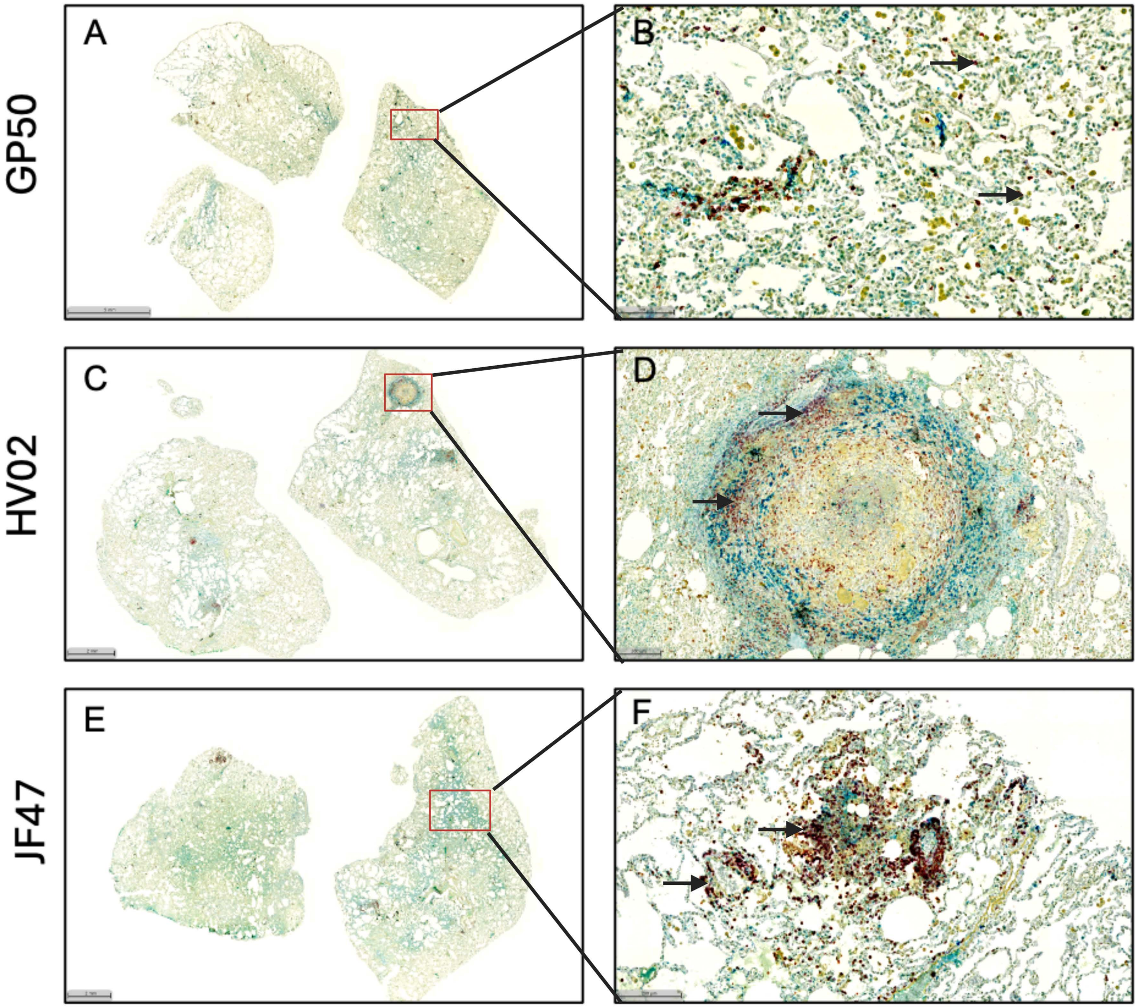
GP50
HV02
JF47
A
B
C
D
E
F

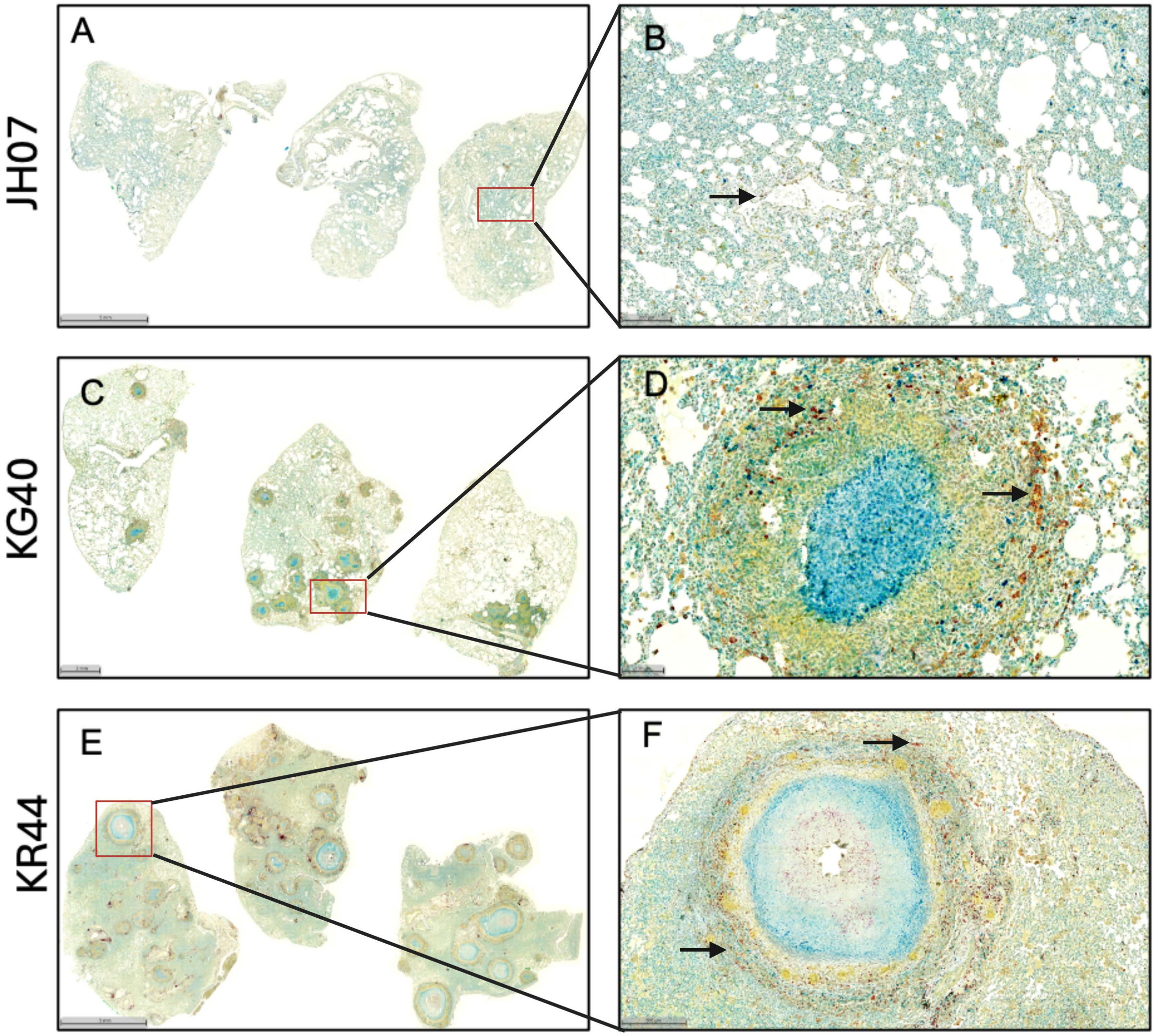
JH07
KG40
KR44
A
B
C
D
E
F

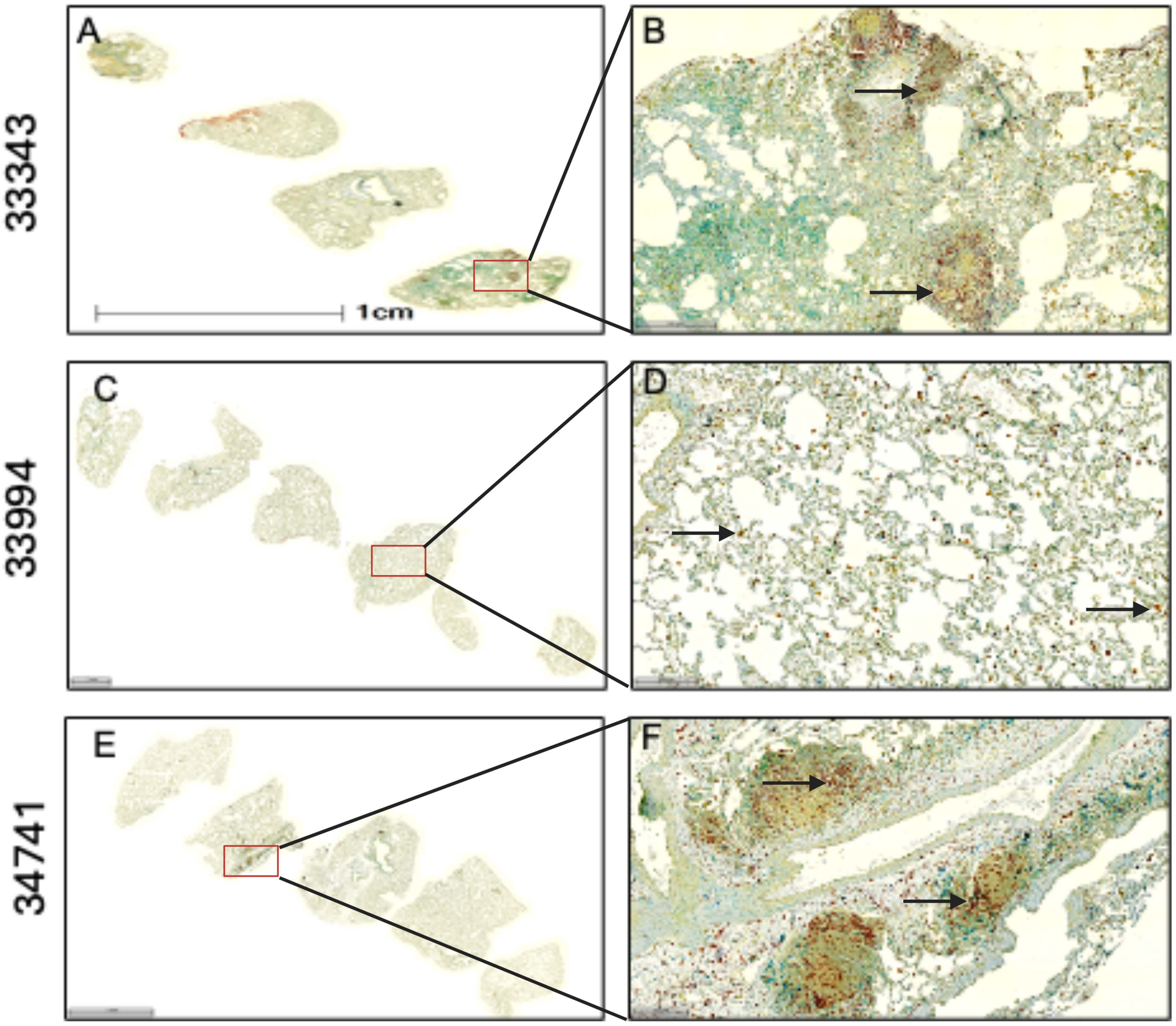

33343
33994
34741
A
B
C
D
E
F
1cm

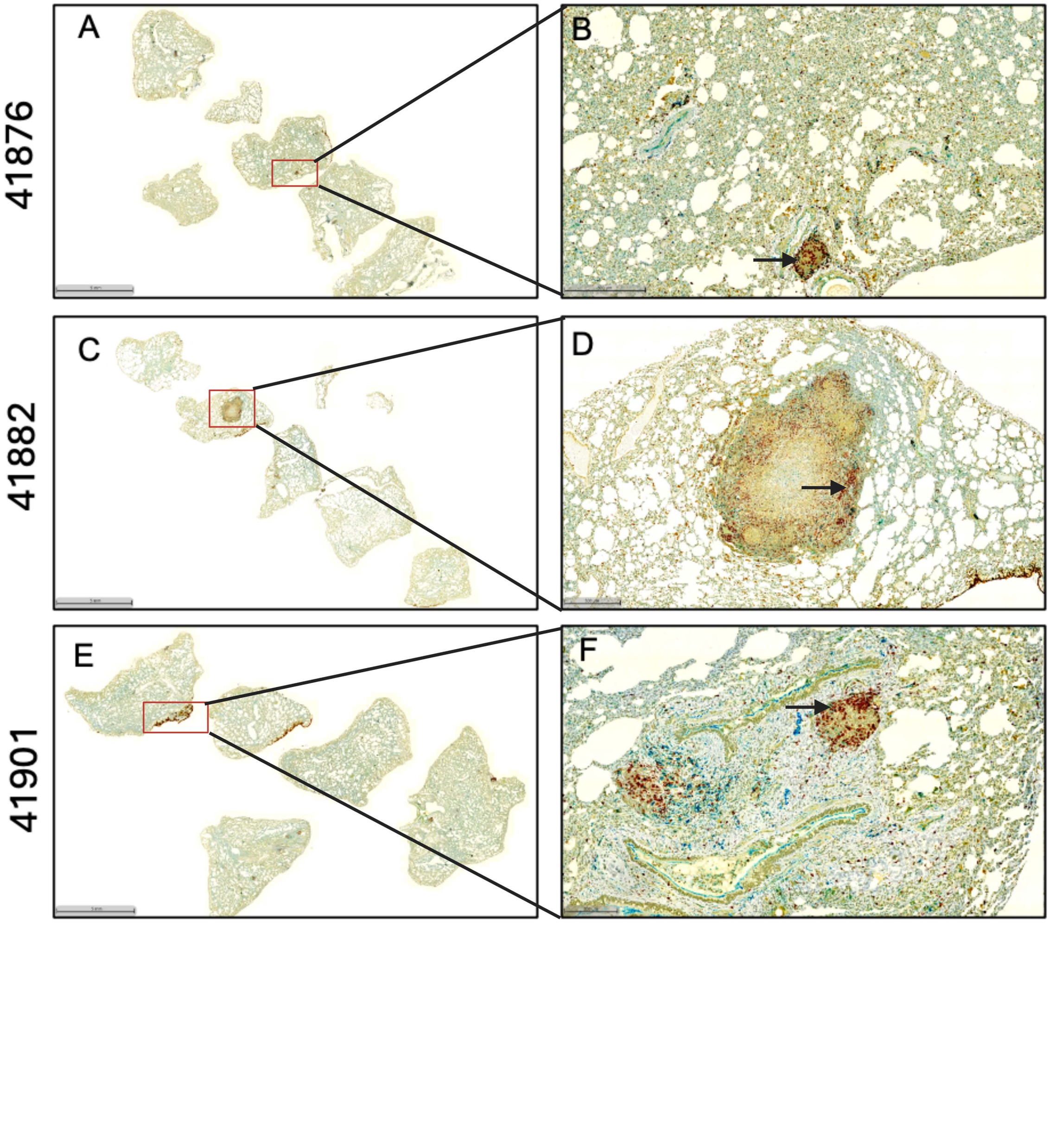
41876
A
B
41882
C
D
41901
E
F

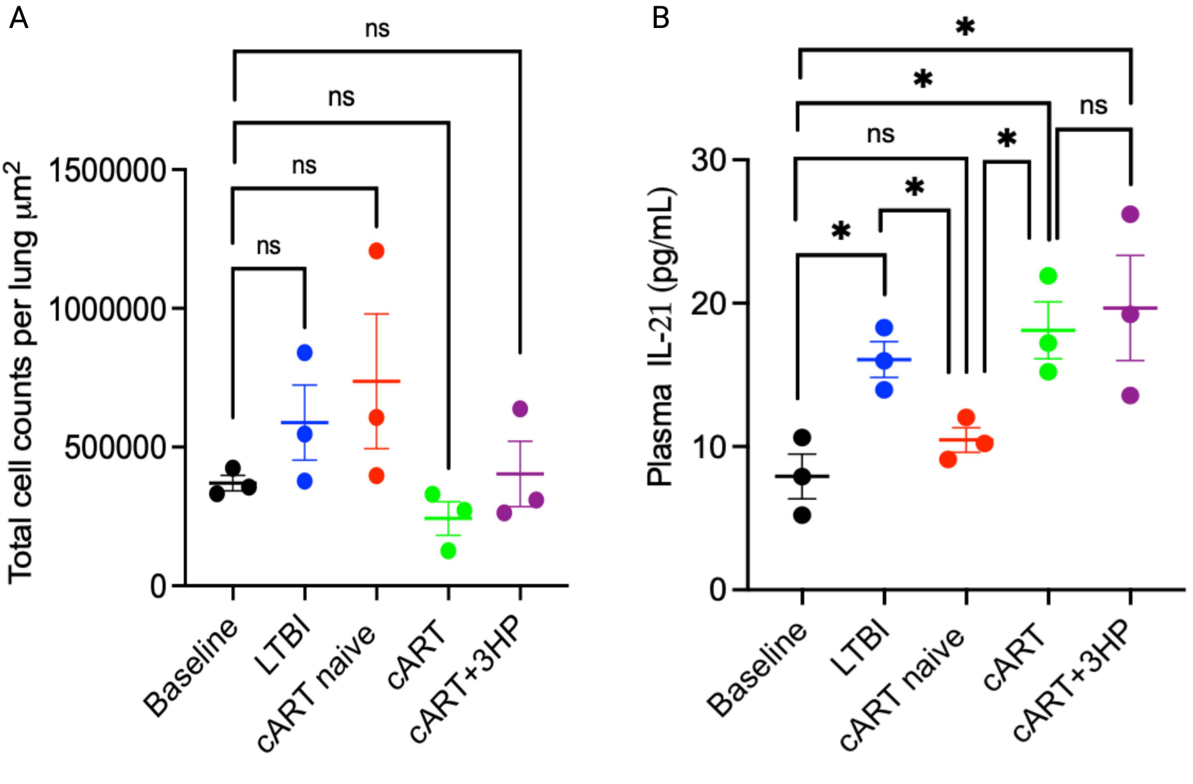
A
ns
ns
ns
ns
Total cell counts per lung μm2
1500000
1000000
500000
0
Baseline
LTBI
cART naive
cART
cART+3HP
B
*
*
ns
ns
*
*
*
30
20
10
0
Plasma IL-21 (pg/mL)
Baseline
LTBI
cART naive
cART
cART+3HP

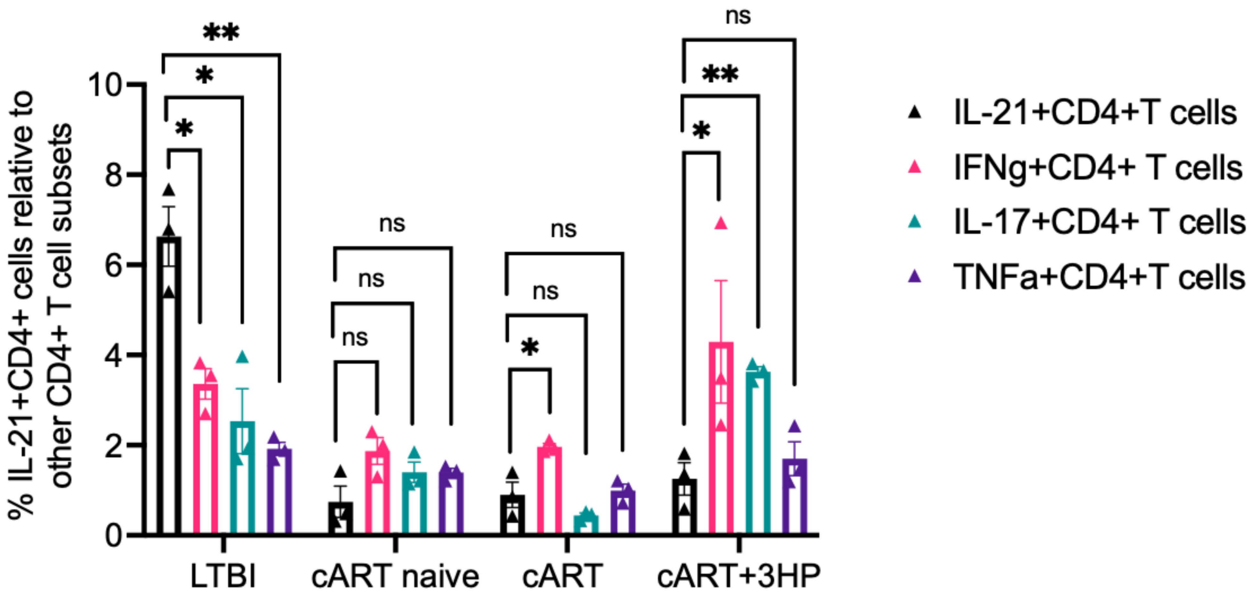

% IL-21+CD4+ cells relative to other CD4+ T cell subsets
0
2
4
6
8
10
**
*
*
ns
ns
ns
ns
ns
*
ns
**
*
LTBI
cART naive
cART
cART+3HP
IL-21+CD4+T cells
IFNg+CD4+ T cells
IL-17+CD4+ T cells
TNFa+CD4+T cells

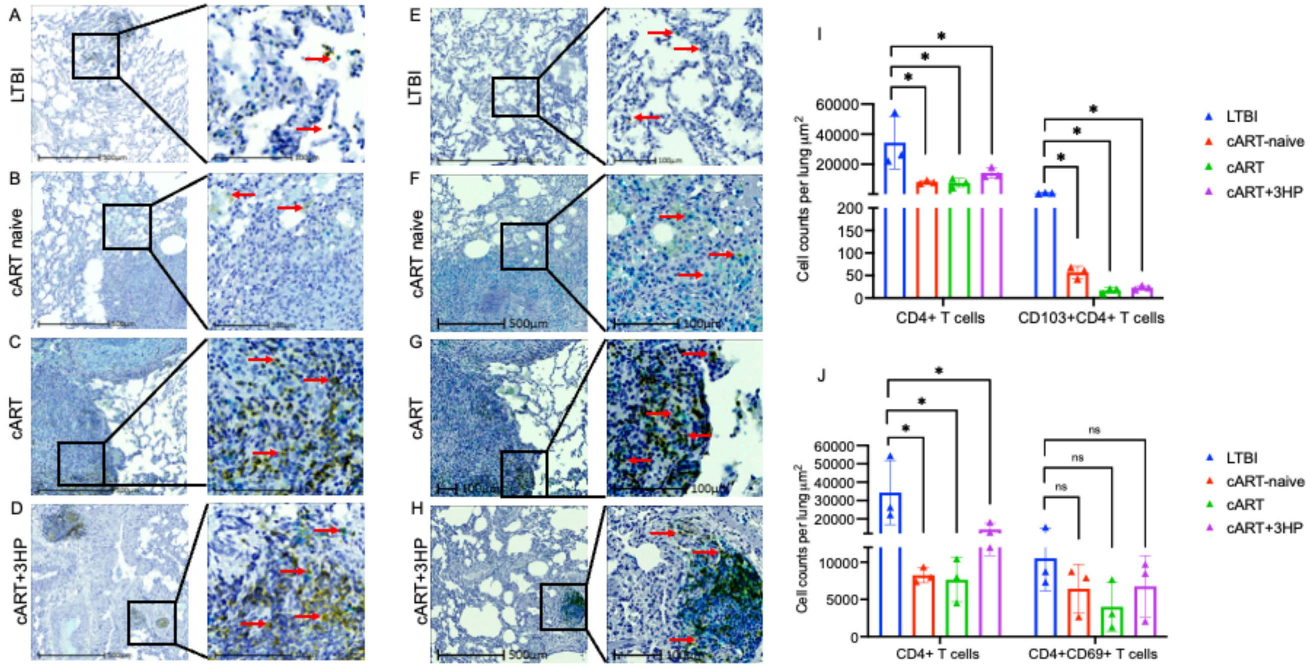
A
B
C
D
E
F
G
H
LTBI
cART naive
cART
cART+3HP
500μm
100μm
I
J
Cell counts per lung μm2
CD4+ T cells
CD103+CD4+ T cells
CD4+CD69+ T cells
LTBI
cART-naive
cART
cART+3HP
*
ns

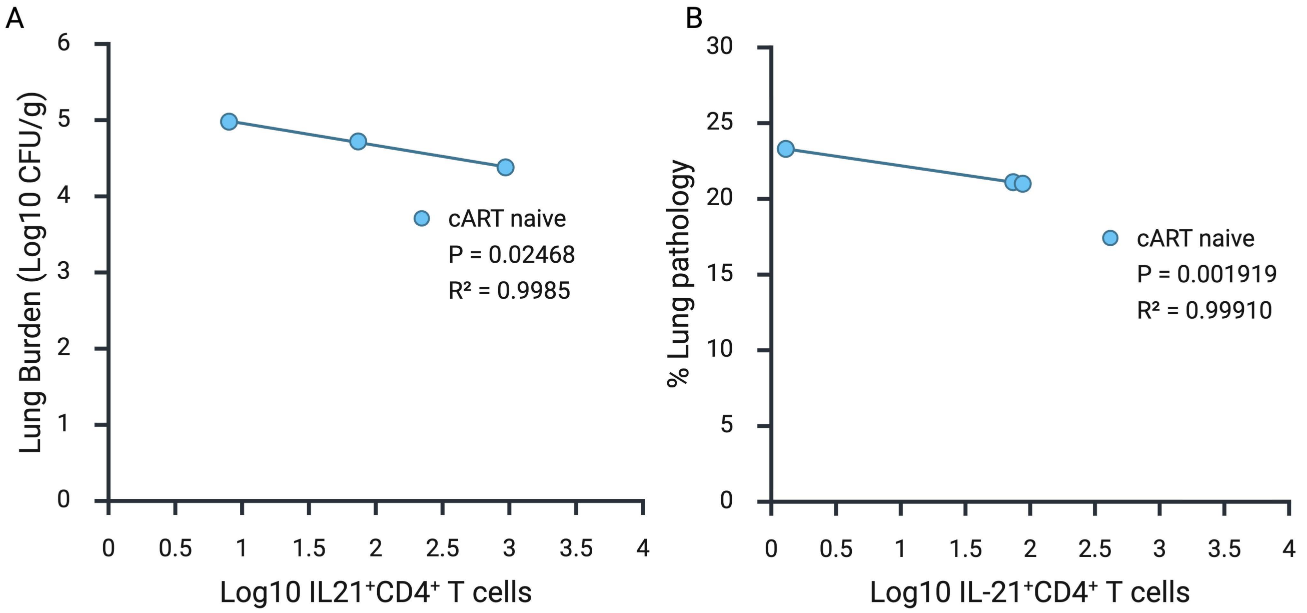

A
Lung Burden (Log10 CFU/g)
6
5
4
3
2
1
0
0
0.5
1
1.5
2
2.5
3
3.5
4
Log10 IL21⁺CD4⁺ T cells
cART naive
P = 0.02468
R² = 0.9985
B
% Lung pathology
30
25
20
15
10
5
0
0
0.5
1
1.5
2
2.5
3
3.5
4
Log10 IL-21⁺CD4⁺ T cells
cART naive
P = 0.001919
R² = 0.99910

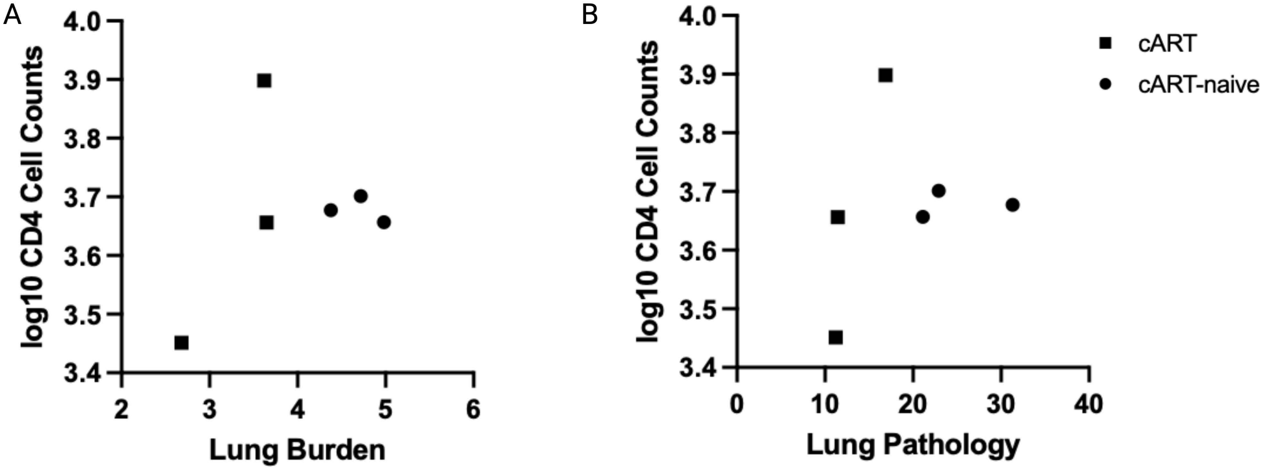

	Lung Burden vs. cART-naive	Lung Burden vs. cART
Pearson r		
r	-0.3906	0.8261
95% confidence interval		
R squared	0.1525	0.6824
P value		
P (one-tailed)	0.3723	0.1906
P value summary	ns	ns
Significant? (alpha = 0.05)	No	No

	Lung Pathology vs. cART-naive	Lung Pathology vs. cART
Pearson r		
r	0.1203	0.9048
95% confidence interval		
R squared	0.01446	0.8187
P value		
P (one-tailed)	0.4616	0.14
P value summary	ns	ns
Significant? (alpha = 0.05)	No	No

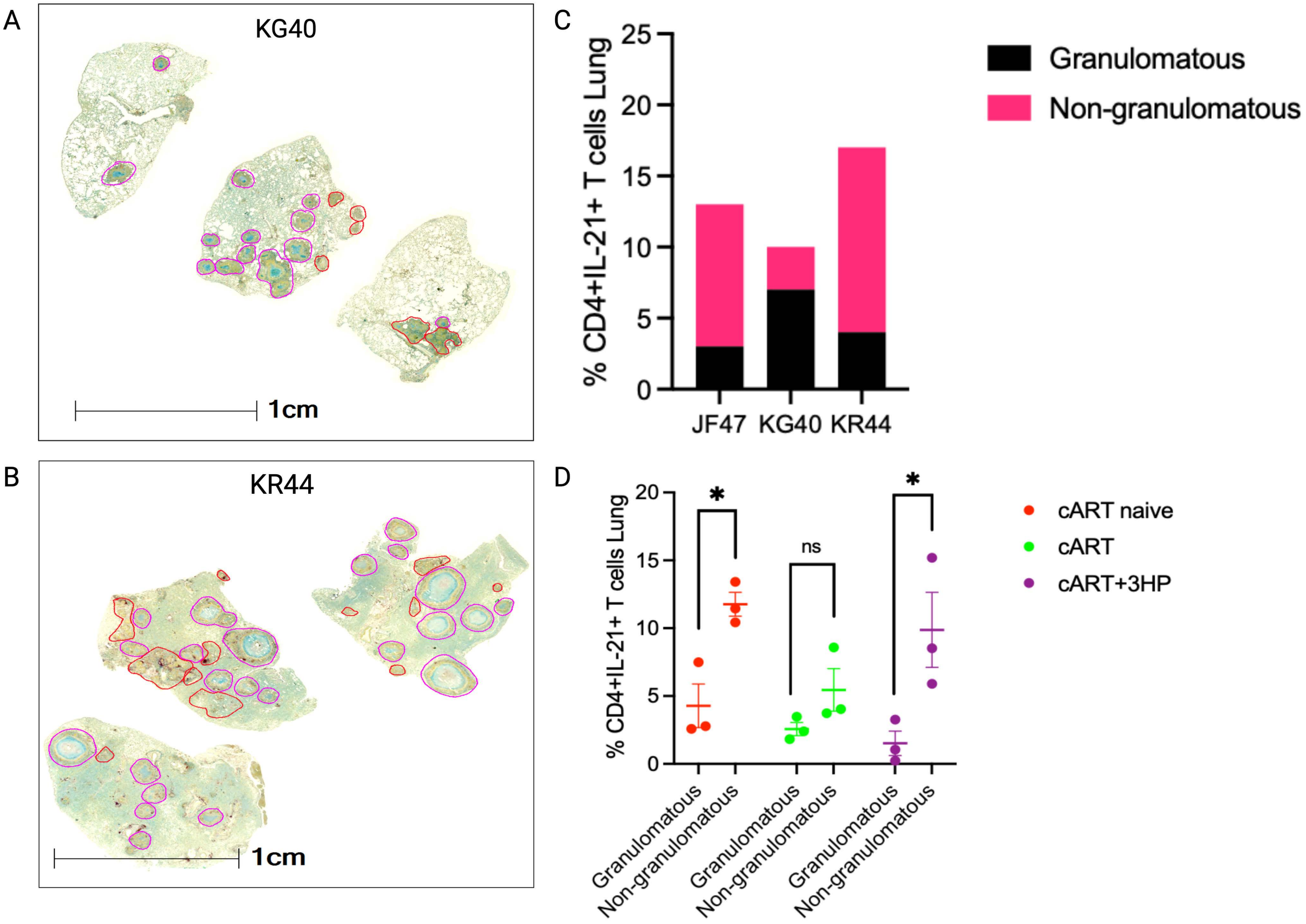

A
KG40
1cm
B
KR44
1cm
C
% CD4+IL-21+ T cells Lung
25
20
15
10
5
0
JF47
KG40
KR44
Granulomatous
Non-granulomatous
D
% CD4+IL-21+ T cells Lung
20
15
10
5
0
*
ns
*
Granulomatous
Non-granulomatous
Granulomatous
Non-granulomatous
Granulomatous
Non-granulomatous
cART naive
cART
cART+3HP

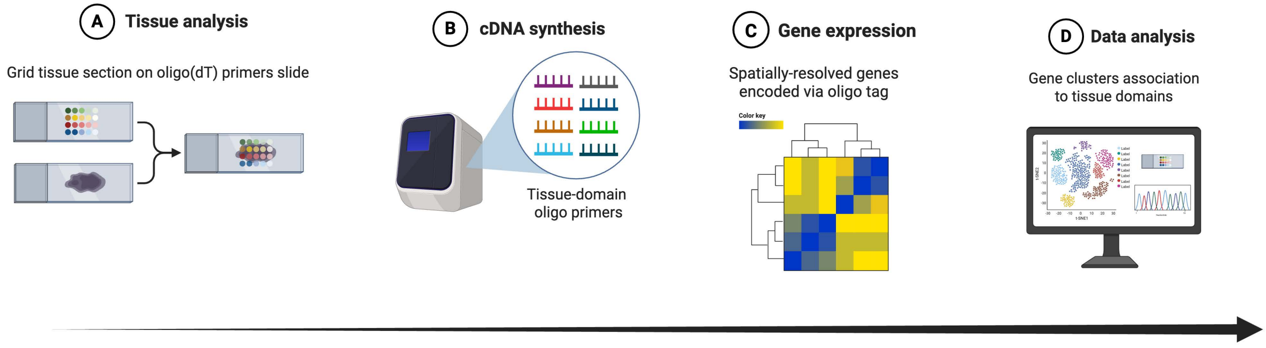

A
Tissue analysis
Grid tissue section on oligo(dT) primers slide
B
cDNA synthesis
Tissue-domain oligo primers
C
Gene expression
Spatially-resolved genes encoded via oligo tag
Color key
D
Data analysis
Gene clusters association to tissue domains

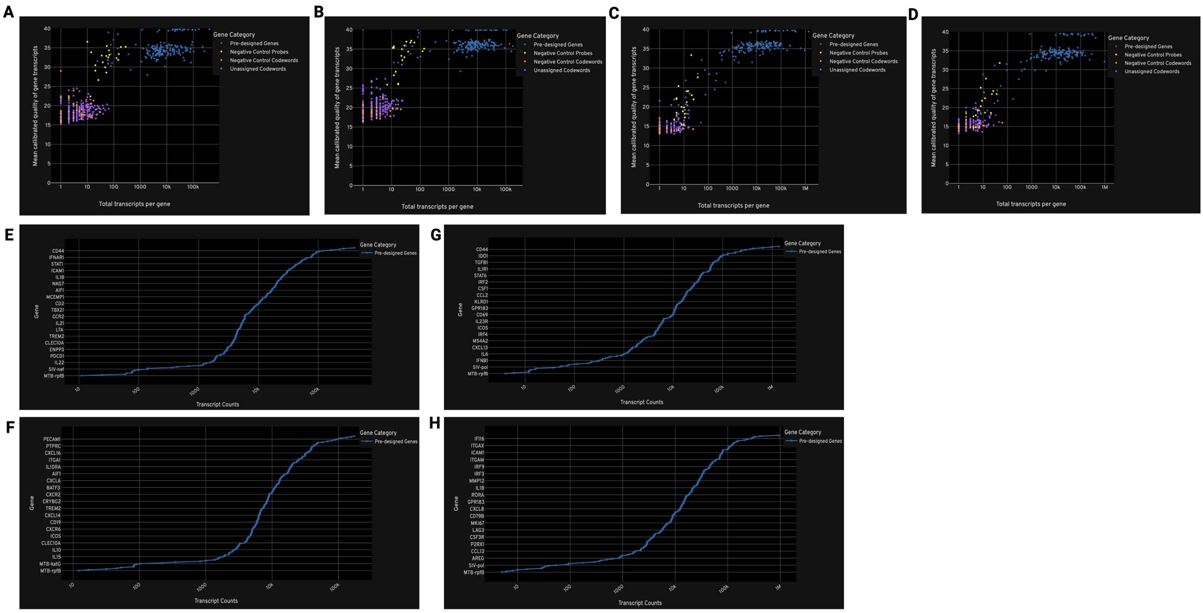

I

	LTBI	cART naïve	cART	cART+3HP
No. of cells detected	347,255	216,156	147,895	118,859
Median transcripts per cell	8	9	24	35
Median genes per cell	6	7	14	18
High quality gene transcripts	95.20%	95.50%	92.00%	87.50%
Total high quality decoded transcripts	4,000,617	2,919,184	8,215,688	7,219,547
Nuclear transcripts per 100 μm^2	25.1	30.2	84.5	113.8
Thickness of high quality decoded transcripts (in μm)	12.741	10.857	6.769	7.513
Cellular transcripts per 100 μm^2	16	18.6	76	89.6

A

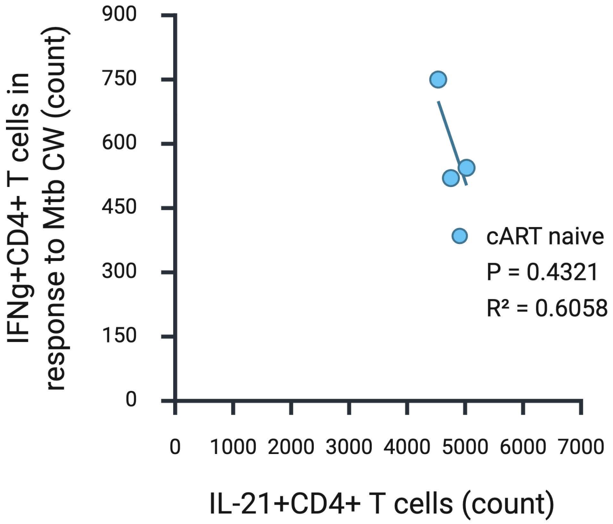

900
750
600
450
300
150
0
IFNg+CD4+ T cells in response to Mtb CW (count)
cART naive
P = 0.4321
R² = 0.6058
0 1000 2000 3000 4000 5000 6000 7000
IL-21+CD4+ T cells (count)

B

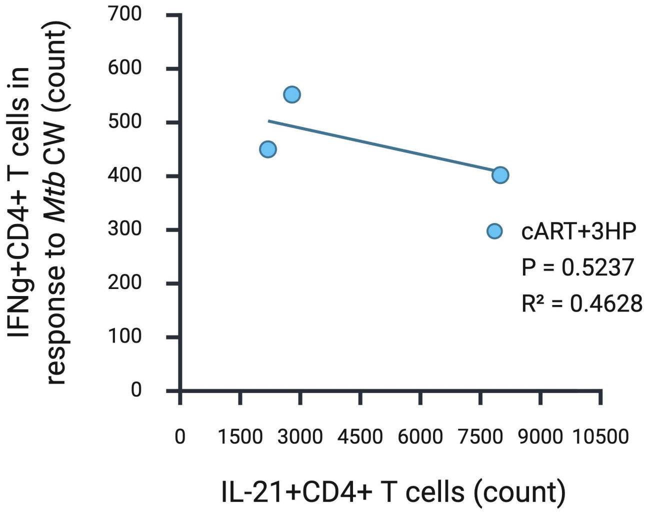

700
600
500
400
300
200
100
0
IFNg+CD4+ T cells in response to Mtb CW (count)
cART+3HP
P = 0.5237
R² = 0.4628
0 1500 3000 4500 6000 7500 9000 10500
IL-21+CD4+ T cells (count)

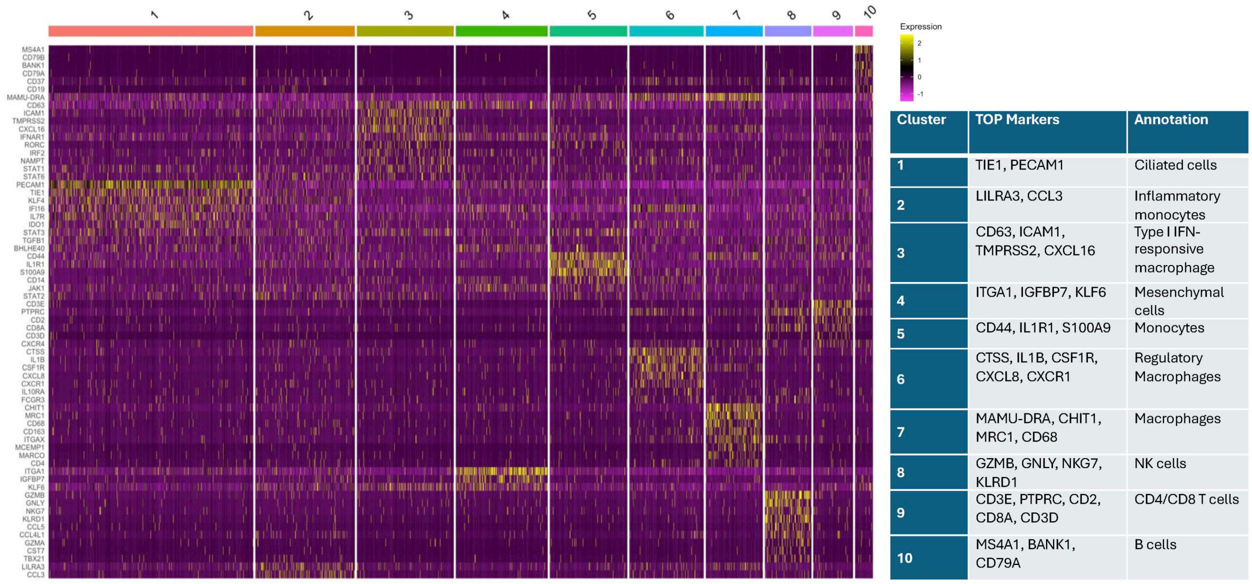

Cluster	TOP Markers	Annotation
1	TIE1, PECAM1	Ciliated cells
2	LILRA3, CCL3	Inflammatory monocytes
3	CD63, ICAM1, TMPRSS2, CXCL16	Type I IFN-responsive macrophage
4	ITGA1, IGFBP7, KLF6	Mesenchymal cells
5	CD44, IL1R1, S100A9	Monocytes
6	CTSS, IL1B, CSF1R, CXCL8, CXCR1	Regulatory Macrophages
7	MAMU-DRA, CHIT1, MRC1, CD68	Macrophages
8	GZMB, GNLY, NKG7, KLRD1	NK cells
9	CD3E, PTPRC, CD2, CD8A, CD3D	CD4/CD8 T cells
10	MS4A1, BANK1, CD79A	B cells

Heatmap of Top Marker Gene Expression by Cluster: LTBI

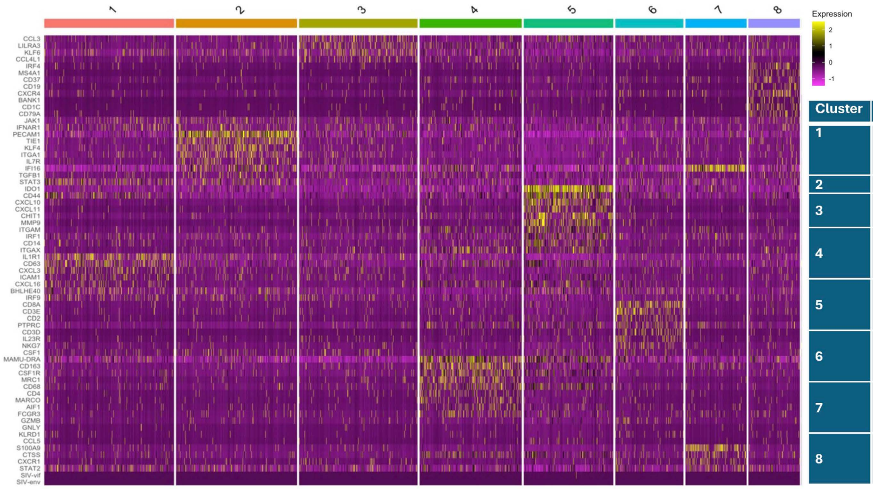

Cluster	TOP Markers	Annotation
1	IL1R1, CD63, CXCL3, ICAM1 STAT3	Pro-inflammatory Monocyte
2	TIE1, PECAM1	Ciliated cells
3	CCL3, LILRA3, KLF6, CCL4L1	Monocytes
4	CD163, MARCO, CD4, CD68 MRC1, CSF1R, AIF1	Macrophages
5	CXCL10, CXCL11, CHIT1, MMP9	Pro-inflammatory Macrophages
6	CD8A, CD3E, CD2, PTPRC, CD3D, IL23R	Th17 $CD4^{+}$ T cells
7	IRF4, BANK1, CD37, CD19, CD79A, MS4A1	B cells
8	GZMB, GNLY, NKG7, KLRD1, CCL5	NK Cells

Heatmap of Top Marker Gene Expression by Cluster: cART naive

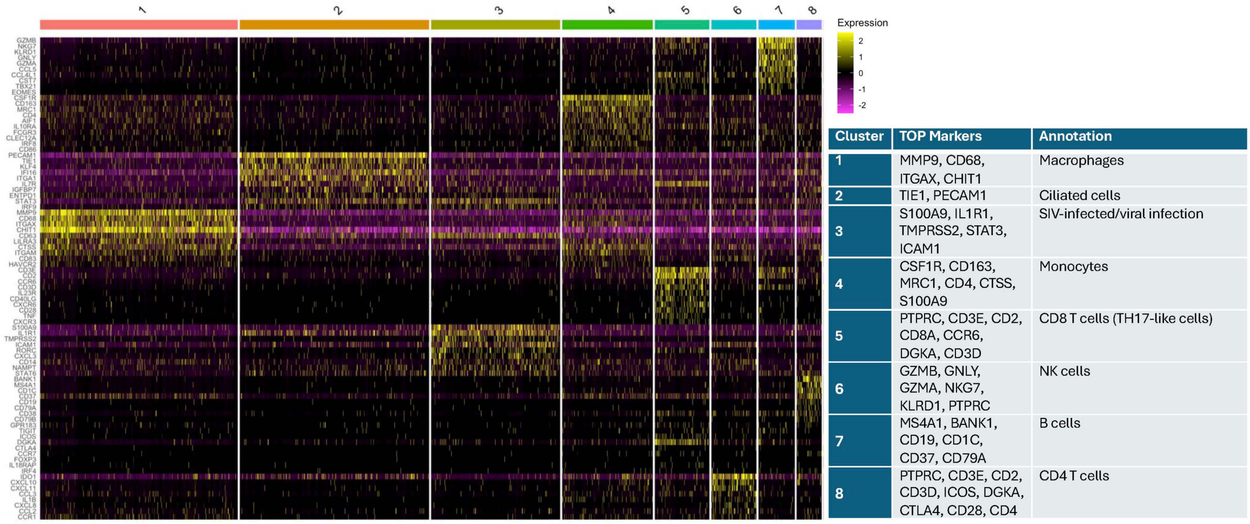

Cluster	TOP Markers	Annotation
1	MMP9, CD68, ITGAX, CHIT1	Macrophages
2	TIE1, PECAM1	Ciliated cells
3	S100A9, IL1R1, TMPRSS2, STAT3, ICAM1	SIV-infected/viral infection
4	CSF1R, CD163, MRC1, CD4, CTSS, S100A9	Monocytes
5	PTPRC, CD3E, CD2, CD8A, CCR6, DGKA, CD3D	CD8 T cells (TH17-like cells)
6	GZMB, GNLY, GZMA, NKG7, KLRD1, PTPRC	NK cells
7	MS4A1, BANK1, CD19, CD1C, CD37, CD79A	B cells
8	PTPRC, CD3E, CD2, CD3D, ICOS, DGKA, CTLA4, CD28, CD4	CD4 T cells

Heatmap of Top Marker Gene Expression by Cluster: cART

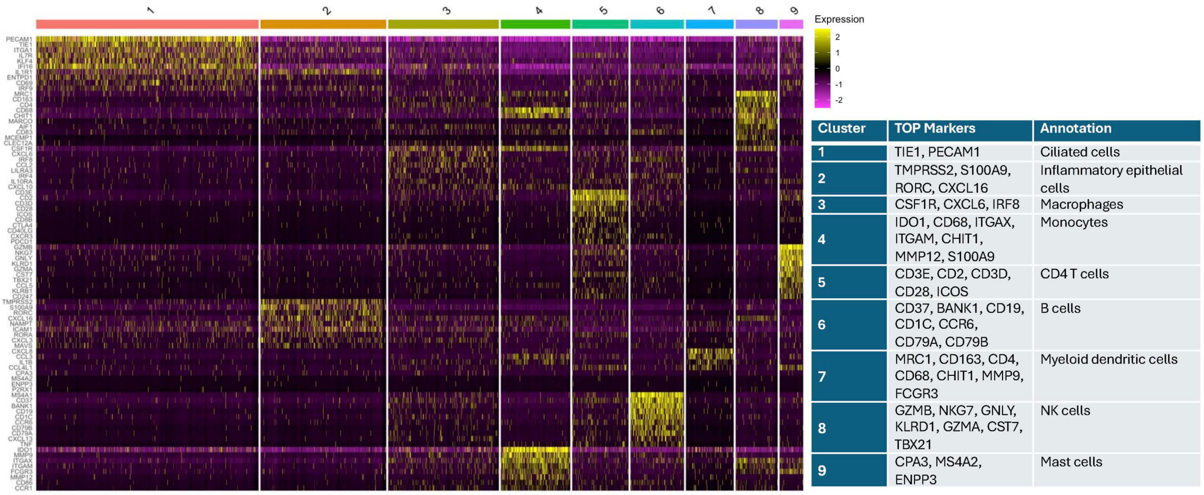

Cluster	TOP Markers	Annotation
1	TIE1, PECAM1	Ciliated cells
2	TMPRSS2, S100A9, RORC, CXCL16	Inflammatory epithelial cells
3	CSF1R, CXCL6, IRF8	Macrophages
4	IDO1, CD68, ITGAX, ITGAM, CHIT1, MMP12, S100A9	Monocytes
5	CD3E, CD2, CD3D, CD28, ICOS	CD4 T cells
6	CD37, BANK1, CD19, CD1C, CCR6, CD79A, CD79B	B cells
7	MRC1, CD163, CD4, CD68, CHIT1, MMP9, FCGR3	Myeloid dendritic cells
8	GZMB, NKG7, GNLY, KLRD1, GZMA, CST7, TBX21	NK cells
9	CPA3, MS4A2, ENPP3	Mast cells

Heatmap of Top Marker Gene Expression by Cluster: cART+3HP

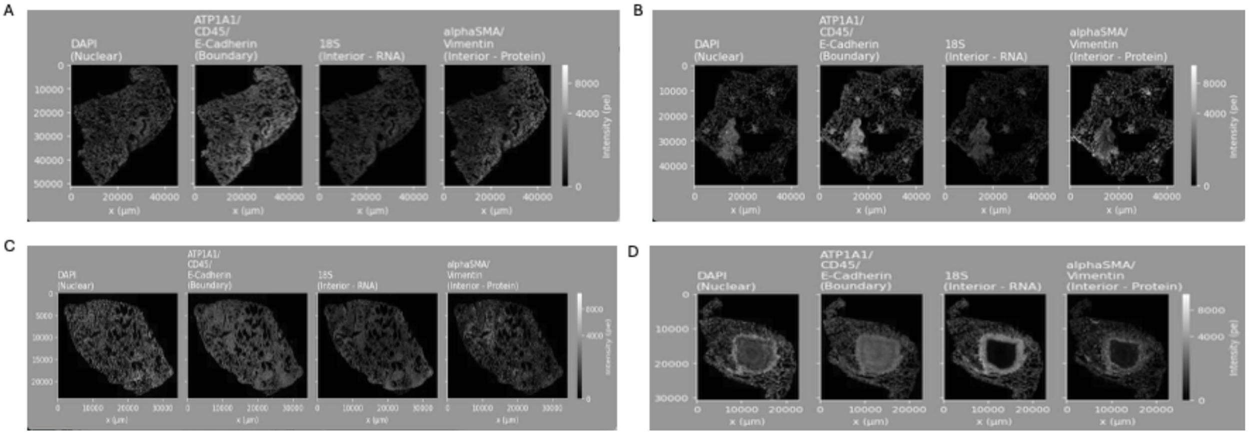

A
B
C
D
DAPI (Nuclear)
ATP1A1/ CD45/ E-Cadherin (Boundary)
18S (Interior - RNA)
alphaSMA/ Vimentin (Interior - Protein)
Intensity (pe)
x (μm)

Sample ID	Group	Description
HV02	LTBI	Baseline (2 regions)
KG40	LTBI + SIV	Untreated (2 regions)
33343	LTBI + SIV + cART	cART treated (3 regions)
41882	LTBI + SIV + cART + 3HP	cART treated + TB therapy (4 regions)

Cell Type	Reference Markers
Macrophage	CD63,MRC1(CD206),CD44,PTPRC(CD45),CD163,CD83, CD14, MNDA, FCGR3(CD16)
Monocyte	MRC1(CD206),CD44,PTPRC(CD45), CD83,ITGAX, CSF3R(CD114),NAMPT, S100A9
CD4 T cells	PTPRC(CD45),CD44,CD3D,CD69,IL23R, CD3E, CD4
CD8 T cells	PTPRC(CD45),CD44,CD3D,CD8B,CD69, CD8A,CD3E
NK cells	PTPRC(CD45),CD44, NCAM1(CD56), NKG2A
B cells	PTPRC(CD45),CD44,CD79B,CD69,CD19, CD79A
Mast cells	ENPP3(CD203c),CD44,KIT(CD117),CD69, PTPRC(CD45)
Myeloid dendritic cells	ITGAM(CD11b), PTPRC(CD45), CDC1
Ciliated cells	TIE1 (CD31), PECAM1 (CD31)

Animal ID	Group	Sex	Age	Therapy initiation	MAMU Type		
					A*01	***B*08***	***B*17***
33343	cART treated	Female	6.30	2 wks post-SIV	Negative	Negative	Positive
33994	cART treated	Male	7.00	2 wks post-SIV	Negative	Negative	Positive
34741	cART treated	Female	5.30	2 wks post-SIV	Negative	Negative	Negative
35974	cART treated	Female	4.40	2 wks post-SIV	Negative	Negative	Positive
34561	cART+3HP treated	Female	7.90	2 wks post-SIV	Negative	Negative	Negative
41898	cART+3HP treated	Male	4.93	2 wks post-SIV	Positive	Negative	Negative
41901	cART+3HP treated	Male	4.88	2 wks post-SIV	Negative	Negative	Negative
41882	cART+3HP treated	Male	4.132	2 wks post-SIV	Negative	Negative	Negative
41876	cART+3HP treated	Male	5.102	2 wks post-SIV	Negative	Negative	Negative
41879	cART+3HP treated	Male	5.61	2 wks post-SIV	Negative	Negative	Negative
KR44	cART naïve	Male	5.50	No cART	Positive	Negative	Negative
LC88	cART naïve	Male	4.10	No cART	Negative	Negative	Negative
JH07	cART naïve	Male	7.32	No cART	Negative	Negative	Negative
JF23	cART naïve	Male	7.26	No cART	Positive	Negative	Negative
KG40	cART naïve	Male	5.51	No cART	Negative	Negative	Negative
IP88	cART naïve	Male	7.05	No cART	Positive	Negative	Negative
JI68	cART naïve	Male	6.06	No cART	Negative	Negative	Negative
JE48	cART naïve	Male	6.33	No cART	Negative	Negative	Negative
GP50	LTBI	Male	11.18	No cART	Positive	Negative	Negative
JF47	LTBI	Male	7.32	No cART	Negative	Negative	Negative
HV02	LTBI	Male	9.34	No cART	Negative	Negative	Negative
JD72	LTBI	Male	7.38	No cART	Negative	Negative	Negative

Lung	Tissue Area (μm^2)
LTBI	233556800
	239154448
	273528896
cART naïve	228770464
	166268784
	153978912
cART	170309040
	107882232
	90442016
cART+3HP	153487856
	139960048
	205737744
